# Identification and Characterization of Robust Hepatocellular Carcinoma Prognostic Subtypes Based on an Integrative Metabolite‐Protein Interaction Network

**DOI:** 10.1002/advs.202100311

**Published:** 2021-07-11

**Authors:** Di Chen, Yiran Zhang, Wen Wang, Huan Chen, Ting Ling, Renyu Yang, Yawei Wang, Chao Duan, Yu Liu, Xin Guo, Lei Fang, Wuguang Liu, Xiumei Liu, Jing Liu, Wuxiyar Otkur, Huan Qi, Xiaolong Liu, Tian Xia, Hong‐Xu Liu, Hai‐long Piao

**Affiliations:** ^1^ CAS Key Laboratory of Separation Science for Analytical Chemistry Dalian Institute of Chemical Physics Chinese Academy of Sciences Dalian 116023 China; ^2^ University of Chinese Academy of Sciences Beijing 100049 China; ^3^ Department of Thoracic Surgery Cancer Hospital of China Medical University Liaoning Cancer Hospital & Institute Shenyang 110042 China

**Keywords:** cancer metabolism, hepatocellular carcinoma, immune regulation, metabolite‐protein interactions, subtype

## Abstract

Metabolite‐protein interactions (MPIs) play key roles in cancer metabolism. However, our current knowledge about MPIs in cancers remains limited due to the complexity of cancer cells. Herein, the authors construct an integrative MPI network and propose a MPI network based hepatocellular carcinoma (HCC) subtyping and mechanism exploration workflow. Based on the expressions of hub proteins on the MPI network, two prognosis‐distinctive HCC subtypes are identified. Meanwhile, multiple interdependent features of the poor prognostic subtype are observed, including hypoxia, DNA hypermethylation of metabolic pathways, fatty acid accumulation, immune pathway up‐regulation, and exhausted T‐cell infiltration. Notably, the immune pathway up‐regulation is probably induced by accumulated unsaturated fatty acids which are predicted to interact with multiple immune regulators like SRC and TGFB1. Moreover, based on tumor microenvironment compositions, the poor prognostic subtype is further divided into two sub‐populations showing remarkable differences in metabolism. The subtyping shows a strong consistency across multiple HCC cohorts including early‐stage HCC. Overall, the authors redefine robust HCC prognosis subtypes and identify potential MPIs linking metabolism to immune regulations, thus promoting understanding and clinical applications about HCC metabolism heterogeneity.

## Graphical abstract

The authors construct an integrative metabolite‐protein interaction (MPI) network and develop a MPI prediction model. The MPI network helps redefine robust hepatocellular carcinoma prognostic subtypes. The subtypes show remarkable differences in hypoxia, DNA methylation of metabolic enzymes, immune pathways, fatty acid accumulation as well as tumor microenvironment. Meanwhile, unsaturated fatty acids are potential links between the metabolism and immune regulations.

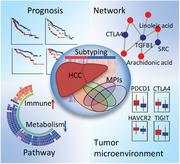

## 1 Introduction

Metabolic reprogramming is one hallmark of cancer.^[^ ^1^ ^]^ Cancer cells need to reprogram their metabolism processes to meet their fast‐growing biomass and energy demands.^[^ ^2^, ^3^ ^]^ Meanwhile, the metabolic alterations can also promote cancer development and progression through certain forms of metabolite and protein interactions (MPIs).^[^ ^4^, ^5^ ^]^ Furthermore, the metabolic reprogramming is highly‐adaptive, alternative MPIs or pathways will be activated in the absence of a certain nutrient or regulator.^[^ ^6^, ^7^ ^]^ Consequently, the metabolic landscape, composing of both metabolites, proteins and interactions between them, is highly‐variable in different cancer types, subtypes, or even the same cancer type under different conditions.^[^ ^8^, ^9^ ^]^ Understanding the metabolic alterations and their relevant adaptive MPIs can provide meaningful insights into cancer metabolism and potential therapeutic targets.

Liver is one of the most important metabolic organs. Hepatocellular carcinoma (HCC) as the most common type of liver cancer undoubtedly displays remarkable alterations in nearly all types of major metabolic processes, like glucose, lipid as well as oxidative metabolism.^[^ ^10^, ^11^ ^]^ The diversity and heterogeneity of HCC metabolic alterations make it difficult to understand the HCC metabolic landscape. Several systematical HCC stratification strategies have been developed to investigate the HCC metabolic heterogeneity. Some meaningful subtypes as well as the metabolic, signaling, and immune characters were recognized,^[^ ^12^, ^13^, ^14^, ^15^ ^]^ for example, ACSS1 was found to be related with HCC tumor growth and malignancy under hypoxia conditions based on a genome scale metabolic (GEM) model of HCC.^[^ ^12^ ^]^ However, these analyses focused particularly on proteins or enzymes rather than metabolites or MPIs which also play key functions in cancers, limiting our understanding about metabolic pathways.

Here, to further explore the metabolism heterogeneity and identify MPIs linking metabolism to other cancer‐associated processes in HCC, we proposed a MPI network based HCC subtyping and mechanism investigation workflow. First, we constructed a directed MPI network. Then, based on the MPI network and multi‐omics data from the cancer genome atlas (TCGA)^[^ ^16^ ^]^ two prognosis‐distinctive HCC subtypes and their corresponding biological and clinical characters were identified. The poor prognostic HCC subtype was associated with remarkable hypoxia, DNA hypermethylation of metabolism enzymes, fatty acid accumulation, and immune cell infiltration. Meanwhile, the poor prognostic subtype can be further divided into two metabolism‐distinctive sub‐populations based on tumor microenvironment (TME) compositions. Notably, consistent results were observed across multiple independent HCC cohorts. Moreover, owing to a MPI prediction model we developed, some accumulated metabolites, especially unsaturated fatty acids (like linoleic acid and arachidonic acid), were identified to interact with some key immune regulators (like SRC and CTLA4) for the poor prognostic subtype. Together, we redefined robust HCC metabolism subtypes, characterized the subtype‐specific clinical and molecular features and highlighted potential MPIs linking cancer metabolism and immune regulations, thus promoting a comprehensive understanding about HCC metabolism and providing guidelines for HCC precision diagnosis and treatment.

## 2 Results

### 2.1 An Integrative MPI Network

**Figure 1:**
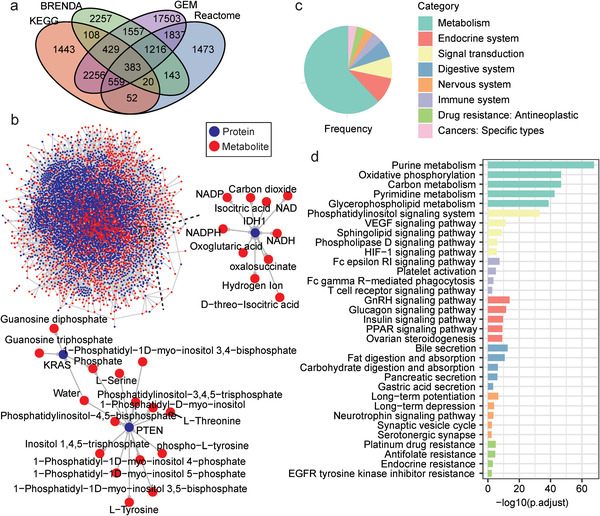
An integrative MPI network. a) Venn plot showing the overlap of MPIs obtained from different resources. b) Sketch of the MPI network. Proteins and metabolites are represented by different colors and links represent interactions. Two sub graphs are enlarged. An edge from one metabolite to one protein means the metabolite is a substrate of the protein, while a reversed direction means the metabolite is a product. Bidirectional edges mean reversible functions. c) Pie plot of the pathway categories the MIPros enriched in. d) The top‐5 enriched pathways in each category. p.adjust: tested by hypergeometric distribution, adjusted by Benjamini and Hochberg method.

We extracted MPIs from four data resources including Kyoto Encyclopedia of Genes and Genomes (KEGG),^[^ ^17^ ^]^ Reactome,^[^ ^18^ ^]^ Human‐GEM,^[^ ^19^ ^]^ and BRENDA^[^ ^20^ ^]^ (**Figure** **1**a). Integrating these MPIs, a directed MPI network including 31236 unique MPIs covering 1870 metabolites and 4132 proteins (represented by the encoding genes) was constructed (Figure 1b and Table S1, Supporting Information). Many well‐known proteins that play crucial roles in cancer like IDH1, PTEN, and KRAS were included in this network (Figure 1b). Most of the metabolite‐interacting proteins (MIPros) belonged to metabolism pathways, some of them also participated in the other aspects like endocrine system, signal transduction, as well as immune system (Figure 1c,d), highlighting that metabolites can participate in various cellular processes via interactions with a variety of proteins.

### 2.2 Pan‐Cancer Investigation Identified HCC Specific Metabolism Alterations

**Figure 2:**
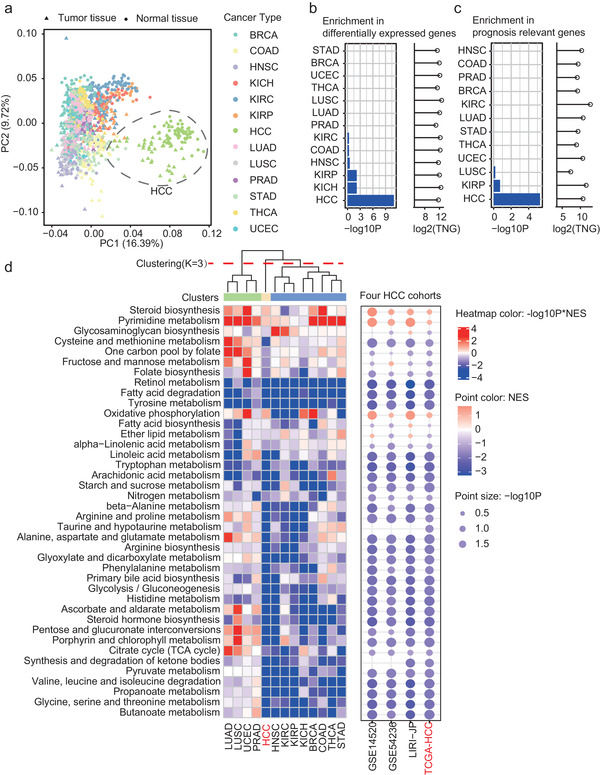
Metabolic alterations between tumor and normal tissues across 13 cancer types. a) PCA projection of paired tumor and normal tissue samples from 13 different cancer types in TCGA. Samples are colored by cancer types. b,c) Enrichment of MIPros in differentially expressed (b) or prognosis relevant genes (c) for different cancers. The differential genes in mRNA expressions between paired tumor and normal tissues were examined by paired Wilcox‐test (significant genes: P adjusted by false discovery rate (FDR) < 0.01 and |log2 transformed fold change (log2FC)| >1). The prognosis relevant genes were examined by Cox proportional hazards model (significant genes: *p* < 0.01). The MIPro enrichment was examined by hypergeometric distribution with Bonferroni correction. −log10P: −log10 transformed *p*‐value; TNG: total number of the differentially expressed genes or prognosis genes. d) The left heatmap shows the metabolic pathway alterations between tumor and normal tissues across 13 cancer types. The right point plot shows the metabolic pathway alterations between tumor and normal tissues across 4 HCC cohorts. NES: normalized enrichment score, p: calculated by GSEA. BRCA: breast invasive carcinoma; COAD: colon adenocarcinoma; HNSC: head and neck squamous cell carcinoma; KICH: kidney chromophobe; KIRC: kidney renal clear cell carcinoma; KIRP: kidney renal papillary cell carcinoma; LUAD: lung adenocarcinoma; LUSC: lung squamous cell carcinoma; PRAD: prostate adenocarcinoma; STAD: stomach adenocarcinoma; THCA: thyroid carcinoma; UCEC: uterine corpus endometrial carcinoma.

Principle component analysis (PCA) was performed on the mRNA expressions of core MIPros (i.e., MIPros with more than four neighbors in the MPI network) across TCGA‐HCC and the other 12 cancer types in TCGA. It was obvious to see HCC showed the most distinctive metabolic patterns (far away from the other cancer types) based on the first two principle components (PCs) from PCA (**Figure** **2**a). Meanwhile, the paired tumor and normal tissues of HCCs and the other cancer types were generally separable (Figure 2a and Figure S1, Supporting Information).

Two kinds of cancer‐relevant genes, that is, differentially expressed or prognosis‐relevant genes (see Experimental Section), were identified for each individual cancer type. By contrast, HCC showed the highest enrichment of MIPros in both kinds of the genes (Figure 2b,c). Moreover, pathway level alterations between tumor and normal tissues in each cancer type were estimated by gene set enrichment analysis (GSEA) (Figure 2d). HCC also displayed the most distinguished metabolic alterations among the 13 cancer types (HCC was clustered into one isolated cluster when the cluster number *K* = 3, Figure 2d). A large fraction of the metabolic pathways were down‐regulated in the HCC tumor tissues compared to the normal ones, as observed across four independent HCC cohorts including the TCGA‐HCC, a Japanese HCC cohort (LIRI‐JP) from international cancer genome consortium (ICGC), and two HCC datasets from gene expression omnibus (GEO), confirming the HCC‐specific down‐regulation of multiple metabolism pathways (Figure 2d).

### 2.3 The MIPro Expression Profiles Defined Two Robust HCC Subtypes Showing Remarkable Differences in Prognosis and Hypoxia

**Figure 3:**
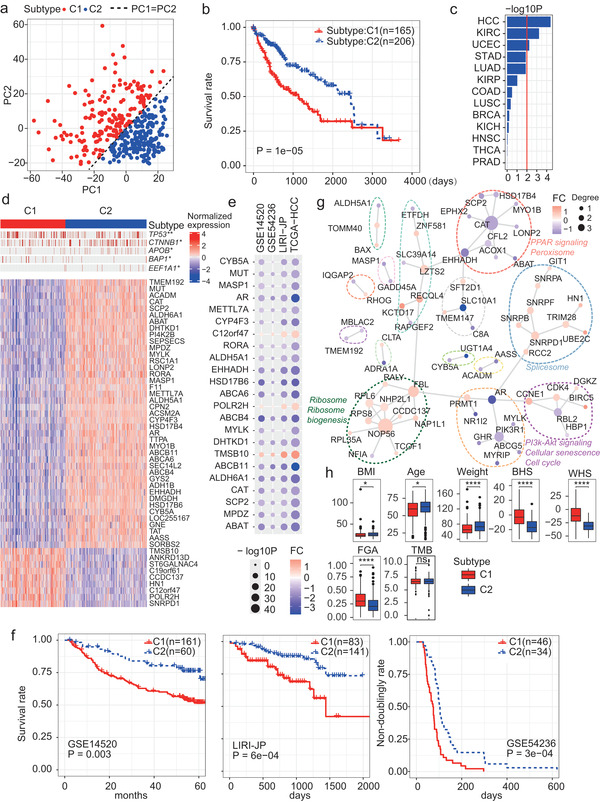
Identification of two stable HCC subtypes. a) PCA projection of TCGA‐HCC tumor samples based on mRNA expressions of core MIPros. Points are colored according to the consensus clustering results, and the two subtypes can be exactly separated by the line PC1 = PC2. b) Kaplan–Meier (KM) plot of the differential prognosis between the two TCGA‐HCC subtypes. c) The subtyping workflow was also applied onto the other cancer types and the corresponding prognosis differences between two subtypes were calculated. The bar length is proportional to −log10P (log‐rank test). d) Heatmap representation of the genomic and transcriptomic differences between the two HCC subtypes. *: *p* < 0.05, **: *p* < 0.01, Fisher exact test on whether the mutation is enriched in one‐specific subtype, one‐sided. e) The top‐ranked subtype‐relevant genes show consistently differential expressions across four HCC cohorts. FC: fold change. P: Wilcox‐test with Bonferroni correction. f) KM plots of the prognosis differences between the two HCC subtypes identified in the other three HCC cohorts. g) PPI network modules of the subtype‐relevant genes. Node colors represent the mRNA level FCs between subtypes C1 and C2. Node sizes are proportional to node degrees. Bigger circles with different colors represent different network modules. Pathways enriched by a module are annotated around the module, with the same color of the corresponding circle. h) Significant differences between the two subtypes in the other clinical or biological factors. The centers of the boxes represent the median values. The bottom and top boundaries represent the 25th and 75th percentiles. The whiskers indicate 1.5 times of the interquartile range. The dots represent points falling outside this range. *: *p* < 0.05, **: *p* < 0.01, ***: *p* < 0.001, ****: *p* < 0.0001, ns: not significant, unpaired Wilcox‐test. The same for the other boxplots.

The above analysis showed the distinctive metabolic features of HCC compared to the other cancer types. Meanwhile, high metabolism heterogeneity also existed within HCC. To characterize the heterogeneity, PCA was utilized to reduce the MIPro expressions into two dimensions, that is, PC1 and PC2, and then consensus clustering method was applied on the first two PCs to cluster the HCCs into two subtypes (see also Experimental Section). As results, the two subtypes can also be partitioned by the line PC1 = PC2 exactly (**Figure** **3**a, PC1 < PC2 for the first subtype C1 and PC1> PC2 for the other subtype C2). Notably, the subtype C1 showed a significantly worse prognosis outcome than C2 (Figure 3b and Figure S2a, Supporting Information). The same analysis was also conducted in the other cancer types. Prognosis subtypes were also identified for KIRC and UCEC (Figure S2b, Supporting Information), whereas HCC was with the most remarkable prognosis differences (Figure 3c).

To characterize the molecular basis of the two HCC subtypes, we recognized somatic mutations specially enriched in one subtype. C1 possessed more mutations in three well‐known tumor suppressors including *TP53* and *BAP1*, while C2 possessed more mutations in genes *CTNNB1, APOB1*, and *EEF1A1* (Figure 3d). Meanwhile, we also defined some subtype‐relevant genes based on their mRNA expressions (see Experimental Section). A large fraction of the top‐ranked subtype‐relevant genes such as *ACADM*, *CAT*, *SCP2*, *AR*, and *ALDH5A1* were down‐regulated for C1 (Figure 3d). We utilized the top‐30 subtype‐relevant genes to build a subtype classifier (see Experimental Section). Applying this subtype classifier on the other independent HCC cohorts, two subtypes with consistent expressional alterations (Figure 3e) and clinical prognostic differences (Figure 3f) emerged.

Moreover, subtype‐relevant PPI network modules were recognized (Figure 3g). Among them, the CAT‐centered module was down‐regulated for C1. Members in this module were mainly peroxisomes (Figure 3g and Figure S2c, Supporting Information) and their interacting metabolites were enriched in multiple metabolic pathways, especially fatty acid relevant pathways (Figure S2d, Supporting Information). Indeed, peroxisomes are closely related with lipid metabolism owing to their ability to conduct fatty acid oxidation and lipid synthesis.^[^ ^21^ ^]^ The peroxisome down‐regulations in C1 implied the inhibition of fatty acid oxidation and lipid synthesis. Another ACADM‐AASS composed module was also down‐regulated, and their interacting metabolites were also enriched by fatty acids or unsaturated fatty acids (Figure S2d, Supporting Information). The NOP56‐centered and SNRPD1‐centered modules were mainly up‐regulated in C1, and their members were mainly involved in ribosome biogenesis and spliceosome (Figure S2c, Supporting Information). Additionally, a large fraction of the subtype‐relevant genes also interacted with the other proteins, like ESR1, LRRK2, NTRK1, JUN, and CUL3 (Figure S2e, Supporting Information), implying the complexity of metabolism interactome landscape.

The proteome level differences between the two subtypes were also examined (Figure S2f, Supporting Information). Although most of the top‐ranked subtype‐relevant genes were down‐regulated in C1 (Figure 3e), some other proteins like Syk and S6 (Figure S2f, Supporting Information) were up‐regulated in C1 (Figure S2g, Supporting Information). These up‐regulated proteins were especially enriched in pathways like HIF‐1, PI3K/AKT, and insulin signaling pathways. HIF‐1 acts as a master mediator of cellular response to hypoxia.^[^ ^22^ ^]^ The HIF‐1 signaling pathway up‐regulation implied a hypoxia condition. Correspondingly, C1 subtype showed remarkably higher hypoxia scores than C2 (Figure 3h, the hypoxia scores BHS and WHS were respectively estimated by Buffa^[^ ^23^ ^]^ and Winter^[^ ^24^ ^]^ methods). Multiple immune relevant pathways like chemokine signaling pathway and natural killer cell mediated cytoskeleton were also up‐regulated in C1.

These two HCC subtypes also showed significant differences in the other aspects. The average body weight of C1 was significantly lower than C2, but the body mass index (BMI) of C1 was only slightly lower than C2, and patients in C1 were a little younger than C2 (Figure 3h). C1 also showed a significant higher fraction of genome alteration (FGA) than C2, whereas the two subtypes did not show differences in tumor mutation burden (TMB). To be noted, these two subtypes did not show significant differences in HBV/HCV infection or liver fibrosis severity (Figure S2h, Supporting Information).

### 2.4 The Poor Prognostic Subtype Was Associated with DNA Methylation of Metabolic Pathways, Up‐regulation in Immune Pathways, and Accumulation of Fatty Acids

**Figure 4:**
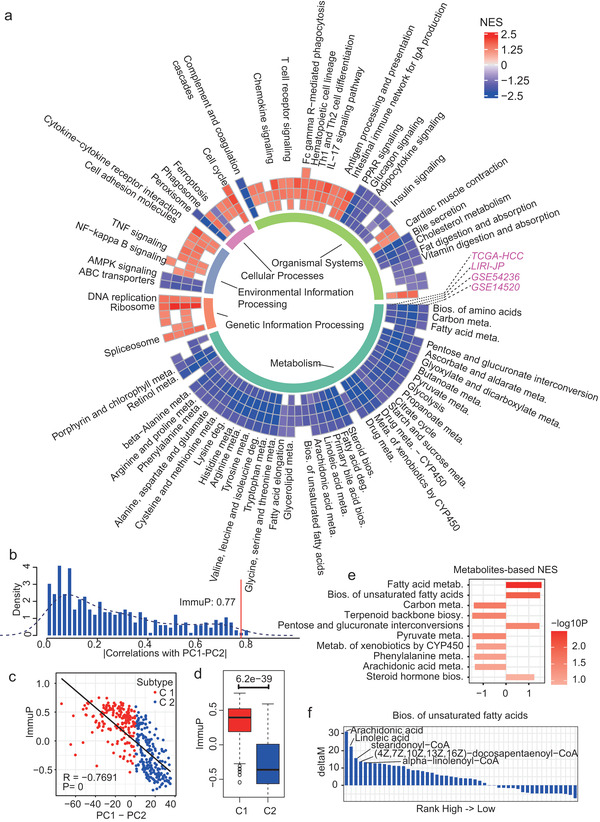
Interplay between metabolism and immune regulations. a) Circos plot of GSEA‐based pathway differences between the two subtypes (from the outmost to the inner layers: results obtained from TCGA‐HCC, LIRI‐JP, GSE54236, and GSE14520). The inner most ring annotates pathway categories. Grids in the outer 4 rings are colored by the GSEA‐based NESs and a positive/negative NES means the pathway is significantly up/down regulated in the poor prognostic subtype C1. A null grid means the pathway difference is not significant (FDR > 0.1). Only pathways showed consistent results in more than two HCC cohorts are included, and pathway names are only displayed for those showed consistent results in three or four cohorts. meta.: metabolism; bios.: biosynthesis; deg.: degradation. b) Histogram about distribution of Spearman correlation coefficients (absolute values) between the subtype discriminant value PC1–PC2 and the summarized profiles of metabolism pathways. Meanwhile, the corresponding result between the summarized immune profile and PC1–PC2 is marked by a red line. c) Point plot showing the correlation between the summarized immune profile and the subtype discriminant value PC1–PC2 in the TCGA‐HCC. d) Boxplot of the summarized immune profiles in the two TCGA‐HCC subtypes. e) Barplot showing the relative ability of the subtype C1 to accumulate (NES > 0) or consume (NES < 0) metabolites in different metabolic pathways compared to the subtype C2. Each metabolite is assigned with an alteration score deltaM which estimates the relative accumulation score of certain metabolite (see Experimental Section). All the metabolites are ranked by the deltaMs, and utilized as the input of metabolites‐based GSEA. The bar length and color respectively stand for the GSEA‐based NES and −log10P. Only the top‐10 significant pathways are shown. f) Barplot of the detailed metabolite alteration scores within the pathway “biosynthesis of unsaturated fatty acids”. Bar length equals the delatM score of one metabolite.

According to the mRNA profiles, most of the metabolism pathways were down‐regulated in the poor prognostic subtype C1 compared to C2, including the glycolysis pathway which was expected to be up‐regulated given the up‐regulation of HIF‐1 signaling and the hypoxia condition^[^ ^22^ ^]^ in C1, and this observation was consistent across four HCC cohorts (**Figure** **4**a). DNA methylation is associated with gene expression silencing. Consequently, we investigated the DNA methylation differences between the two subtypes. As results, most of the mRNA level down‐regulated pathways for C1, especially the metabolic pathways, as well as other metabolism‐related pathways like AMPK and PPAR signaling pathways (Figure 4a), were hyper‐methylated in C1 compared to C2 (Figure S3a, Supporting Information), suggesting the down‐regulation of metabolism pathways in C1 may be caused by hyper‐methylation of metabolic enzymes, like the lipid metabolic enzymes SCP2, ACSL1, etc., (Figure S3b, Supporting Information), and glucose metabolic enzymes CPT2, GYS2, and SLC2A2 (also known as GLUT2) (Figure S3c, Supporting Information).

The cross‐datasets pathway results also showed consistent up‐regulations of various immune pathways like T cell receptor signaling pathway, Fc gamma R‐mediated phagocytosis and IL‐17 signaling pathway (Figure 4a). Meanwhile, a summarized immune profile (ImmuP, see Experimental Section) showed a strong negative correlation (Figure 4b,c) with the metabolic profile PC1–PC2, and the subtype C1 showed remarkably high ImmuP than C2 (Figure 4d). However, the up‐regulations of these immune pathways cannot be explained from the DNA methylation level alterations (Figure S3a, Supporting Information). There must be other mechanisms linking the metabolic alterations and immune regulations.

In addition, we also utilized the directed MPI network to estimate metabolite alterations in different subtypes. Taken linoleic acid as an example, we extracted the directed MPI sub‐graph for linoleic acid, and evaluated the C1 subtype's relative linoleic acid accumulation according to the expressional differences of the connecting proteins between C1 and C2 subtypes. Since the MIPros (CYP2A13, CYP2B6, CYP1A1, etc.) which can consume linoleic acid were remarkably down‐regulated in C1, but the MIPros (CEL, ACOT7, ACOT4, etc.) catalyzing the productions of linoleic acid were either up‐regulated or slightly down‐regulated, the C1 subtype samples were more likely to accumulate linoleic acid than C2 (Figure S3d, Supporting Information, accumulation score deltaM > 0, see also Experimental Section). Similarly, metabolites involved in fatty acid metabolism and biosynthesis of unsaturated fatty acids pathways like arachidonic acid and linoleic acid were more likely to be accumulated by C1, whereas metabolites involved in the carbon, pyruvate metabolism, or terpenoid backbone biosynthesis pathways were more likely to be consumed (Figure 4e,f and Figure S3e, Supporting Information). To validate the results, we utilized another published HCC cohort where transcriptomics and metabolomics were measured simultaneously.^[^ ^14^ ^]^ We used the transcriptomics data to identify the C1 and C2 subtypes, and tested which metabolites showed significant differences between the two subtypes based on the metabolomics. Among the differential metabolites, glucose and mead acid (an unsaturated fatty acid) respectively showed higher and lower levels in the C1 subtype than C2 (Figure S4a, Supporting Information), consisting with the above estimations that metabolites involved in carbon metabolism/biosynthesis of unsaturated fatty acids were mainly down‐/up‐regulated in C1 compared to C2 (Figure 4e). Meanwhile, the significant low levels of multiple bile acid biosynthesis relevant metabolites in C1 (glycocholate, taurocholate, glycochenodeoxycholate, and taurochenodeoxycholate) were also consistent with the estimations that they were more likely to be down‐regulated or consumed in the C1 subtype (Figure S4b, Supporting Information, deltaM < 0).

Notably, unsaturated fatty acids have been found to promote HCC proliferation and progression,^[^ ^25^ ^]^ in line with the accumulation of unsaturated fatty acid in the poor prognostic subtype. Given the cell cycle pathway was significantly up‐regulated for the subtype C1 (Figure 4a), we speculated the fast‐growing cancer cells of the hypoxia subtype C1 may depend on the accumulated metabolites especially unsaturated fatty acids, rather than metabolites generated by glycolysis, to keep survival, as revealed by recent discoveries.^[^ ^26^ ^]^ Additionally, we also hypothesized the accumulated metabolites (Figure 4f) may play certain roles in the up‐regulations of immune pathways for HCC.

### 2.5 A MPI Prediction Model Helps Identify Potential MPIs Linking Metabolism and Immune Regulations

**Figure 5:**
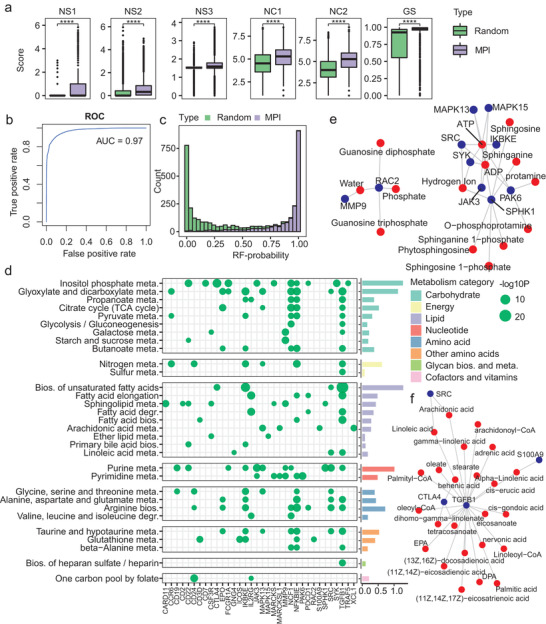
Prediction of MPIs as links between metabolism and immune regulations. a) Boxplot showing the differences between MPIs and randomly combined metabolite‐protein pairs in terms of six network‐based association features. b) ROC curve of the MPI prediction model. c) Histogram of the predicted probabilities of the MPIs and random controls from an independent validation set. d) Interactions between the up‐regulated immune‐relevant proteins and various metabolism pathways. A point represents the predicted interacting metabolites of one protein are significantly enriched in the corresponding metabolism pathway (*p* < 0.05, hypergeometric distribution), and the points are colored by −log10P. The right bars represent the average value in −log10P among all listed immune relevant proteins and are colored by pathway categories. e) Sub‐graph of the up‐regulated immune relevant proteins in the MPI network. Red and blue nodes represent metabolites and proteins respectively. f) High‐confidential MPIs predicted for several up‐regulated immune‐relevant proteins.

To test the hypothesis that the accumulated metabolites in C1 may induce the up‐regulation of immune pathways by interacting with relevant proteins, we developed a MPI prediction model to help identify MPIs between metabolism pathways and immune system. This prediction model utilized six network‐based association features to describe the interactions between metabolites and proteins (**Figure** **5**a, see also Experimental Section). It was obvious to see metabolites and proteins that interact with each other showed significantly higher associations than random pairs (Figure 5a). Accordingly, we developed a well performed random forest (RF)‐based MPI prediction model (Figure 5b, the area under receiver operating characteristic [ROC] curve for the prediction model was 0.97) and the MPIs and random metabolite‐protein pairs can be mostly separated by the predicted probabilities (Figure 5c).

Owing to this MPI prediction model, we identified some high‐confidential MPIs (predicted probability > 0.9) that linked the immune system and metabolism pathways (Figure 5d and Table S2, Supporting Information). Without the predictions, these proteins were only known to interact with very limited metabolites like ATP, ADP, and water (Figure 5e). Among the prediction results, multiple proteins involved in immune system and up‐regulated in C1 were identified to interact with metabolites across various aspects, especially the carbohydrate and lipid metabolism (Figure 5d). For example, CTLA4, a well‐known immune checkpoint^[^ ^27^ ^]^ that can keep T cells from attacking other cells was predicted to interact with metabolites involved in biosynthesis of unsaturated fatty acids and inositol phosphate metabolism. PDCD1 (also known as PD1), another immune checkpoint,^[^ ^28^ ^]^ was identified to interact with arginine biosynthesis or fatty acid degradation and elongation. SRC, a protein tyrosine kinase participating in the regulations of various signaling and immune processes,^[^ ^29^ ^]^ was predicted to interact with multiple metabolic pathways like purine and amino acid metabolism as well as biosynthesis of unsaturated fatty acids. IKBKE, a kinase controlling inflammatory responses can interact with even more metabolic pathways, especially the biosynthesis of unsaturated fatty acids. Of note, the biosynthesis of unsaturated fatty acids, which showed relatively high accumulation for the C1 subtype (Figure 4e), was also with a relatively high connection with these immune regulators (Figure 5d). Focusing on metabolites engaging in this pathway, SRC, CTLA4, IKBKE, and TGFB1 (a critical inducer of epithelial‐to‐mesenchymal transition (EMT) and involved in immune function as well) was found to interact with various fatty acids and coenzyme A (CoAs) (Figure 5f and Figure S5a, Supporting Information). Some of these predicted MPIs have already been reported by previous studies. Linoleic acid has been found to activate TGFB1^[^ ^30^ ^]^ and SRC.^[^ ^31^ ^]^ Arachidonic acid was reported to play a potential role for induction of EMT by interaction with TGFB1.^[^ ^32^ ^]^ The omega‐6 fatty acid (i.e., gamma‐linoleic acid) was also found to play a certain role in TGFB1 up‐regulation.^[^ ^33^ ^]^ Together, these results confirmed the hypothesis that the accumulated metabolites, especially the unsaturated fatty acids, may induce the up‐regulation of multiple immune pathways.

To further understand the systematical functions of the up‐regulated immune pathways, we recognized the PPI network modules among the significantly up‐regulated immune relevant proteins. Multiple PPI network modules emerged, where the SRC‐centered module was the most essential one, that is, with the largest number of connecting clusters (Figure S5b, Supporting Information), highlighting the importance of SRC as well as the SRC relevant MPIs for the poor prognostic subtype. The members from the SRC‐centered network module were enriched not only in immune relevant pathways like Fc gamma R−mediated phagocytosis and chemokine signaling pathway, but also other pathways such as Estrogen signaling pathway and ErbB signaling pathway (Figure S5c, Supporting Information). Besides, the up‐regulations of immune relevant proteins for C1 were also observed in the other HCC cohorts (Figure S5d, Supporting Information). SRC has been found to play an important role in regulating the movement and infiltration of immune cells into tumor cells, and its activation can be mediated by inflammatory cytokines within the tumor microenvironment (TME),^[^ ^34^ ^]^ suggesting the two HCC subtypes may also display differences in tumor microenvironment.

### 2.6 The Two Subtypes also Displayed Remarkable Differences in TME

**Figure 6:**
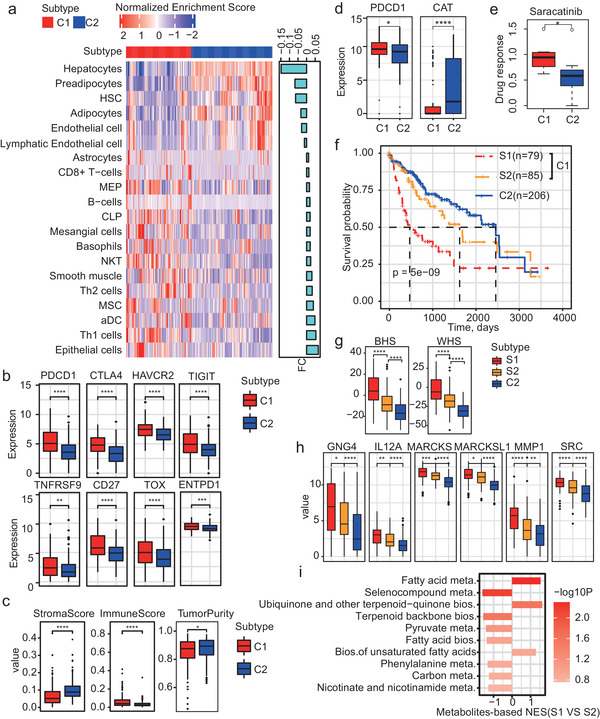
TME characters of the HCC subtypes. a) Heatmap of the potential TME compositions of the TCGA‐HCC samples. The right bars annotate the difference values of the mean cell type enrichment scores between the C1 and C2 subtype. b) Boxplot of the expressions of eight well‐known markers for the CD8+ T cells in the two TCGA‐ HCC subtypes. c) Boxplot of the estimated stroma score, immune score, and purity in the two TCGA‐HCC subtypes. d) Boxplot of the expressions of PDCD1 and CAT in exhausted CD8+ T cells. The single cell RNA‐seq data was obtained from GSE98638, and the cells were classified into C1 and C2 by the HCC subtype classifier. e) Boxplot showing the responses of the liver cancer cell lines to the drug Saracatinib. The information of various liver cancer cell lines was from CCLE and was also classified into C1 and C2 by the HCC subtype classifier. f) KM plot of the differential prognosis among three HCC subtypes in TCGA. C1 was further divided into S1 and S2 based on the TME compositions of the samples. g) Boxplot showing the differences among the three HCC subtypes in TCGA in terms of hypoxia. h) Boxplot showing the differences in multiple immune relevant proteins among the three HCC subtypes in TCGA. i) Barplot showing the ability of the subtype S1 to accumulate (NES > 0) or consume (NES < 0) metabolites in different metabolism pathways compared to the subtype S2. Only the top‐10 significant pathways are shown.

We applied xCell,^[^ ^35^ ^]^ a web‐tool which can predict the enrichment degrees of 64 different cell types among TME based on RNA‐seq data of tissue samples, to estimate the TME compositions of the HCC samples. As results, there were remarkably fewer hepatocytes, preadipocytes, adipocytes, and hematopoietic stem cells (HSCs) but more epithelial cells, activated dendritic cells (aDCs), mesenchymal stem cells (MSCs), smooth muscle cells, and various immune cells like Type 1 T helper (Th1), Type 2 T helper (Th2), natural killer T (NKT), and CD8+ T cells in the C1 subtype than the C2 subtype (**Figure** **6**a). In summary, there might be more immune cell infiltrations in the C1 subtype and this may be related with the up‐regulation of SRC.^[^ ^34^ ^]^ Meanwhile, multiple exhausted T cell markers including PDCD1, CTLA4, HAVCR2, TIGIT, TNFRSF9, CD27, TOX, and ENTPD1^[^ ^36^ ^]^ showed significantly higher expressions in C1 (Figure 6b), indicating the infiltrated T cells in the C1 subtype were mainly exhausted T cells.^[^ ^37^ ^]^ The T cell exhaustion in C1 might be caused by the hypoxic condition.^[^ ^38^ ^]^ Besides, the integrated stroma score of the C1 subtype was less than C2, while the immune score and tumor purity were much higher in C1 (Figure 6c).

To evaluate whether the immune cells also displayed similar metabolism patterns from a single cell perspective, we classified the HCC T cells from a single cell dataset (GSE98638) into the C1‐ and C2‐subtype‐like cells based on the RNA‐seq data. We found C1‐subtype like CD8+ T cells were also with significant higher expressions in PDCD1 and lower expressions in the peroxisome CAT (Figure 6d), confirming the connections between immune response and CAT‐relevant metabolic regulation from a single cell perspective. Moreover, we also identified the C1/C2‐subtype‐like liver cancer cell lines from cancer cell line encyclopedia (CCLE, see also Supporting Methods, Supporting Information) and examined which drugs can generate significant different effects between the two subtypes, and we observed that the C1‐like liver cancer cell lines were more sensitive to a SRC inhibitor saracatinib (also known as AZD0530) than the C2‐like cell lines (Figure 6e).

Since the TME compositions fluctuated a lot in the C1‐subtype samples (Figure 6a), the C1 subtype was further divided into two sub‐populations, termed as S1 and S2, based on the TME profiles. Interestingly, S1 showed the worst prognosis, C2 still showed the most favorable prognosis, and S2 was in the medium (Figure 6f, P = 5e‐9). Meanwhile, the pathway analysis showed various metabolic pathways especially the fatty acid metabolism pathways were down‐regulated in the mRNA level for S1 compared to S2 (Figure S6a, Supporting Information). The three subtypes showed gradually and significantly decreased levels in both hypoxia as well as multiple immune relevant proteins (SRC, MMP1, IL12A, etc., Figure 6g,h), thus providing potential therapeutic targets for the S1 subtype. Meanwhile, the S1 subtype was also more likely to accumulate unsaturated fatty acids compared to S2 (Figure 6i). We also compared the three re‐defined subtypes to two previous HCC subtyping methods including the Hoshida's method^[^ ^39^ ^]^ and the multi‐omics based iCluster method.^[^ ^40^ ^]^ Some associations existed between our subtyping and the previous methods. The S1 subtype was more enriched in the Hoshida‐HS2 subtype (Fisher exact test, P = 3.53e‐17), the S2 subtypes were more enriched in the Hoshida‐HS2 (P = 7.267e‐06) and iCluster1 (P = 1.204e‐05), while the C2 subtype was enriched in the Hoshida‐HS3 (P = 2.057515e‐21, Figure S6b,c, Supporting Information). However, the re‐defined subtypes showed a more significant survival difference than the Hoshida and iCluster subtypes (Figure 6f and Figure S6d,e, Supporting Information).

Moreover, the further divided sub‐populations and their prognosis differences were also validated by the other HCC cohorts (Figure S6f, Supporting Information). It is worth noting that the two‐step subtyping can also be applied to predict HCC progression, where the S1‐subtype HCC patients were most likely to double the HCC tumor size (Figure S6g, Supporting Information, GSE54236) and the S1‐like cirrhosis patients were most likely to progress into HCC (Figure S6g, Supporting Information, GSE15654), S2 was the next, and C2 showed the most favorable outcome. These significant metabolism, immune, and clinical differences across the three subtypes further highlight that the close interactions between fatty acid metabolism and the SRC‐centered immune regulations can promote HCC development and progression.

### 2.7 Validation of Metabolic and Immune Features of the Two Main HCC Subtypes in HCC Cells

**Figure 7:**
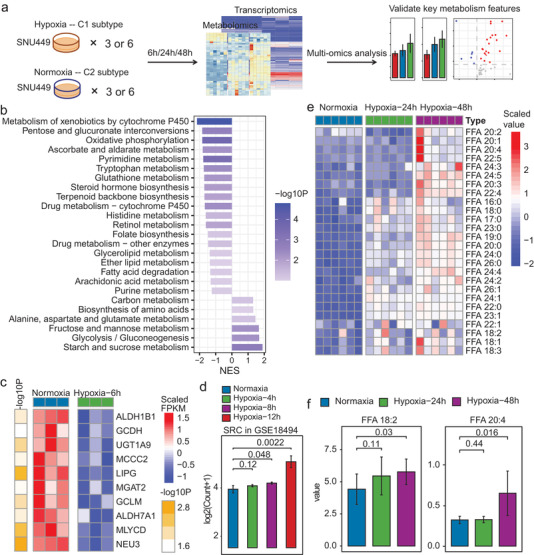
Validation of the two main HCC subtype features. a) Sketch about the experimental analysis. b) GSEA based metabolism pathway differences between the SNU449 cell lines cultured under 6 h of hypoxia and normoxia conditions (*n* = 3 for each condition, P and NES were calculated by GSEA, and only pathway with FDR < 0.3 were shown). c) Heatmap of multiple genes involved in metabolism pathways and showing lower mRNA expressions in the hypoxia group. d) Barplot showing the mRNA expressions of SRC in HepG2 cells under different treatments (*n* = 3 for each condition). e) Heatmap of multiple fatty acids that showing higher levels under hypoxia conditions. The display fatty acids were with *p* < 0.05 for comparing 24/48 h hypoxia group to the normoxia group. Full metabolite names are listed in Table S3, Supporting Information. f) Barplot showing the levels of FFA 18:2 and FFA 20:4 in SNU449 cells under different treatments (*n* = 6 for each condition). P in (c–f): one‐sided T‐test, check on whether one item is less/greater in the hypoxia group. Bar in (d) and (f): mean ± standard deviation.

Considering the C1 subtype showed remarkably higher hypoxia scores than C2 (Figure 3h, BHS, WHS), we utilized SNU449 cell lines cultured under hypoxia and normoxia conditions to mimic the C1 and C2 subtypes, where both transcriptomics and metabolomics profiles were examined to validate the features of the two HCC subtypes (**Figure** **7**a). The expressions of HIF1*α* significantly increased in the SNU449 cells with hypoxia treatment compared to normoxia condition (Figure S7a, Supporting Information). Based on the transcriptomics data, various metabolism pathways were down‐regulated in the hypoxia group (Figure 7b). Meanwhile, multiple immune system pathways were up‐regulated in the hypoxia group (Figure S7b, Supporting Information). For instance, various metabolic enzymes like ALDH1B1, GCDH, and LIPG were significantly down‐regulated (Figure 7c), and IKBKE, NFKB1, RBPJ, etc., which were involved in Th1 and Th2 cell differentiation, were up‐regulated under hypoxia condition (Figure S7c, Supporting Information). Based on another published transcriptomics dataset of hypoxia HCC cell lines (GSE18494),^[^ ^41^ ^]^ similar and even more significant metabolism or immune system pathway alterations were observed in HepG2 cells under 12 hours (h) of hypoxia treatment (Figure S7d, Supporting Information). Meanwhile, the expression levels of SRC, which was predicted to interact with certain unsaturated fatty acids and play a key role for the immune regulation of the C1 subtype (Figure 5f and Figure S5b, Supporting Information), also increased significantly with the hypoxia treatment in the HepG2 cells (Figure 7d).

From the perspective of metabolomics, since various fatty acids especially some unsaturated fatty acids like linoleic acid and arachidonic acid were predicted to be accumulated in the C1 subtype (Figure 4f), we utilized lipidomics profiling to examine the free fatty acid (FFA) alterations. As results, the hypoxia SNU449 cells displayed higher levels in multiple fatty acids (Figure 7e) including various unsaturated ones like FFA 18:2 and FFA 20:4 (Figure 7f, linoleic acid and arachidonic acid are respectively the most common form of FFA 18:2 and FFA 20:4, see Table S3, Supporting Information, for descriptions of the FFAs), and the alterations became more pronounced in a time dependent manner (Figure 7d and Figure S7e and Table S4, Supporting Information).

These results validate that the poor prognosis C1 subtype of HCC is closely related with down‐regulation of enzymes in various metabolism pathways, accumulated fatty acids as well as multiple up‐regulated immune regulators.

## 3 Conclusion

Metabolic reprogramming is not only the result of certain oncogenic changes, but can also promote cancer development and progression through complicated interactions with a tumor ecosystem.^[^ ^42^ ^]^ In this point, we constructed an MPI network to help understand cancer metabolism from a more comprehensive perspective and recognize some meaningful MPIs which can influence other cancer relevant processes. Through a pan‐cancer investigation, we found HCC showed the most distinctive metabolism pattern compared to the other common cancer types. Then, we utilized the MPI network to redefine HCC subtypes and multi‐omics data were integrated with the MPI network to help reveal the latent mechanism of the different HCC subtypes.

Interestingly, the poor prognostic subtype C1 was significantly down‐regulated in nearly all types of metabolism pathways compared to C2. The associations between HCC poor prognosis and down‐regulation in multiple metabolism enzymes have also been recognized in previous studies.^[^ ^13^, ^43^, ^44^ ^]^ However, the reason remains unclear. Here, we found that the down‐regulation of metabolism pathways was largely caused by DNA hyper‐methylation in various metabolic enzymes. Furthermore, we also inferred that the down regulation dominant metabolic enzyme alterations might lead to the accumulation of multiple fatty acids in the subtype C1 which was also related with hypoxia and up‐regulated cell cycle. This is consistent with recent discoveries that constitutively growing cells will depend on unsaturated fatty acids for survival under hypoxia conditions.^[^ ^26^ ^]^

Notably, the two metabolic HCC subtypes also displayed significant differences in immune system. Unlike the metabolic pathways, the immune pathway alterations were independent of DNA methylations. Instead, various MPIs were identified as the potential causes. Multiple metabolites, especially some unsaturated fatty acids were predicted to interact with the immune regulators. Some studies have also reported the unsaturated fatty acids like linoleic acid and arachidonic acid can activate these immune regulators.^[^ ^30^, ^31^, ^32^, ^33^ ^]^ Accordingly, we speculate that several immune regulators showing higher expressions in the C1‐subtype such as SRC, CTLA4, and PDCD1 might serve as C1‐subtype‐specific therapeutic targets.

The metabolism differences may also result in distinctive TME, where the C1‐subtype specific metabolic alterations were associated with immune cell infiltration, especially T‐cell infiltration. T cell infiltration has shown positive prognostic impacts on multiple cancer types, like colorectal,^[^ ^45^ ^]^ ovarian,^[^ ^46^ ^]^ and breast cancers.^[^ ^47^ ^]^ Here, however, the T cell infiltration linked to a poor prognostic subtype of HCC. We found the reason may lie in that the hypoxia condition of C1 leads to T cell exhaustion.^[^ ^38^ ^]^ Moreover, the TME helped further divide the C1 subtype into two sub‐populations which also showed remarkable differences in metabolism, immune regulation, hypoxia, and prognosis. Thus, our study finally redefined three prognostic HCC subtypes which showed distinctive metabolic, immune, and TME characters.

Some previous studies have also identified meaningful HCC subtypes.^[^ ^12^, ^13^, ^14^, ^15^, ^39^, ^40^, ^42^, ^43^, ^44^ ^]^ However, they rarely considered metabolites and MPIs, let alone the interplay among metabolism, immune response, and TME. In this study, we not only re‐defined the HCC subtypes based on a MPI network, but also illuminated the potential MPI‐based regulation mechanisms where immune system and TME were also involved in. Furthermore, the HCC subtyping was consistently proved by multiple independent HCC cohorts, and the prognosis differences were in remarkably significant levels. These HCC cohorts were from different countries and with different races, implying a wide range applicability of the redefined subtypes. Meanwhile, the subtyping can also be applied onto early‐stage HCC or cirrhosis to help predict HCC progression. In‐vitro experimental analysis validated the down‐regulation dominating metabolism enzyme alterations, accumulation of fatty acids, and up‐regulations of multiple immune regulators of the C1 subtype to some degree. Moreover, further experimental investigations will be performed in the future to explore and validate more detailed regulatory mechanisms.

Overall, we proposed an effective HCC subtyping strategy and revealed MPIs linking the metabolite alterations to immune responses and TME. These results illustrated the HCC subtyping relevant molecular mechanism, identified potential drug targets, predicted meaningful MPIs and highlighted the important role of MPIs in HCC, thus can provide guidance for HCC subtyping and promote the precision diagnosis and treatment of HCC.

## 4 Experimental Section

### Cell Culture and Hypoxia Treatment

SNU‐449 cell lines were obtained from American Type Culture Collection. The SNU‐449 cells were cultured in RPMI‐1640 medium (meilunbio, China) with 10% fetal bovine serum (PAN, Germany), and cultivated in a humidified incubator containing 5% CO_2_ at 37 °C. For hypoxia treatment, cells were maintained in a hypoxia chamber (Nuvair, USA) under the atmosphere composition of 1% O_2_, 5% CO_2_, and 94% N₂.

### RNA Extraction and Transcriptomics Profiling

The SNU449 cell lines treated under normoxia and hypoxia for 6 h were collected and their RNAs were isolated using TRIzol reagent. Then, the transcriptomics profiling was performed by RNA‐Seq analysis based on an Illumina Novaseq 6000 system. The gene expressions were estimated by fragments per kilobase of transcript per million mapped reads (FPKM) values. See Supporting Information for more details.

### Lipid Extraction and Fatty Acids Analysis

SNU449 cell lines cultured under normoxia for 24 h and under hypoxia conditions for 24 or 48 h were collected. Liquid–liquid extraction was performed as described previously^[^ ^48^ ^]^ with slight modifications. FFA profiling was carried out on an ACQUITY Ultra Performance Liquid Chromatography (UPLC) system (Waters, Milford, MA, USA) coupled with AB Sciex tripleTOF 5600 plus mass spectrometer (Applied Biosystems Sciex, Foster City, CA, USA). See Supporting Information for more details.

### MPI Network Construction

The MPI network was constructed based on MPIs extracted from four resources including KEGG, Reactome, Human‐GEM, and BRENDA. Edge directions were determined according to whether the metabolites were substrates or products in reactions. See Supporting Information for more details.

### PPI Network Construction

PPIs were obtained from BioGrid, MENTHA, and IntAct. Only PPIs belonged to human were retained and unduplicated PPIs from these databases were integrated to construct the final human PPI network. See Supporting Information for more details.

### Pancancer Data Collection and Analysis

TCGA pancancer RNA‐seq data (normalized by the FPKM and FPKM upper quartile methods) together with the clinical information were downloaded from the genomic data commons website (https://gdc.cancer.gov/about‐data/publications/pancanatlas) and only 13 cancer types of which there were at least 20 paired tumor and normal samples were retained. The RNA‐seq data were log2‐transformed and genes with zero standard deviations were removed.

To find potential relevant genes for each cancer type, differentially expressed genes between paired tumor and normal samples were examined by Wilcox‐test (paired) and log2FCs between tumor and normal samples in terms of mean mRNA expressions were calculated. Meanwhile, prognosis relevance of each gene was estimated by a Cox proportional hazards regression model of the survival R package.^[^ ^49^ ^]^

For PCA‐based projection, the expressional matrix for the paired tumor and normal samples in these 13 cancer types were first scaled by Z‐score. Then, PCA was performed on the expressional matrix, and all samples were projected onto the two dimensions determined by the first two PCs.

Metabolism pathway differences were also examined. For each cancer type, all the genes in the expressional matrix were ranked by log2FCs between tumor and normal tissues and the ranked gene list was utilized as the input for the KEGG‐based GSEA analysis. The GSEA analysis was performed by the clusterProfiler R package. Pathway information was obtained from KEGG.

### HCC Data Collection

For the TCGA‐HCC cohort, the authors obtained the mRNA expression, mutation as well as clinical data from the TCGA pan‐cancer atlas (https://gdc.cancer.gov/about‐data/publications/pancanatlas). The preprocessed DNA methylation data, reverse phase protein array (RPPA)‐based proteomics data,^[^ ^50^ ^]^ and additional clinical information were obtained from cBioportal (https://www.cbioportal.org/).

As comparison, the authors also downloaded the mRNA expressional profiles and clinical information of two HCC cohorts from GEO (https://www.ncbi.nlm.nih.gov/geo/, GSE14520,^[^ ^51^ ^]^ GSE54236),^[^ ^52^ ^]^ and a Japanese HCC cohort from ICGC (project code: LIRI‐JP, https://dcc.icgc.org/projects/LIRI‐JP). Besides, the information for an early stage liver cirrhosis cohort was downloaded from another GEO dataset (GSE15654).^[^ ^53^ ^]^ Meanwhile, matched transcriptomics and metabolomics data for a Thailand HCC cohort were obtained from GEO (GSE76297) and the Supporting Information of the study.^[^ ^14^ ^]^ The information for these collected cohorts were summarized in **Table** **1**. All the HCC‐cohort mRNA matrixes were normalized by Z‐score. Transcriptomics data of hypoxia‐treated HepG2 cell lines were downloaded from GEO (GSE18494).

**Table 1:** Sample information of the HCC cohorts

Character	TCGA‐HCC	GSE14520	GSE54236	LIRI‐JP	GSE15654	GSE76297
Sample No.	371	247	81	232	216	61
Country	USA	China	Italy	Japan	USA	Thailand
Men, No. [%]	68%	85%	79%	74%	54%	Not reported
Age(SD)	60(13)	51(11)	Not reported	67(10)	59(5)	Not reported

### MIPros‐Based HCC Subtyping

Node degrees were calculated based on number of linked neighbors on the MPI network. Metabolites with degrees larger than 200 (e.g., water, hydrogen Ion, ADP, etc., regarded as common metabolites) were removed from the MPI network. Simultaneously, the remained isolated MIPros were also removed. Hereafter retained MIPros with degrees larger than 4 were taken as the core MIPros. PCA was performed on the mRNA expressional profiles of these core MIPros of HCC tumor samples, and consensus clustering analysis (basic clustering method: partitioning around medoids, maxK = 10, by the ConsensusClusterPlus R package^[^ ^54^ ^]^) was performed based on the first two PCs calculated from PCA. The final cluster number was set as K = 2. The PCA‐based step helps obtain the main variations of various MIPros by low dimension PCs and the consensus clustering step helps obtain a robust clustering result.

### Identification of Subtype‐Relevant Genes

Since the mRNA expression data were the most comprehensive among the multi‐omics data, the authors examined the contribution of each gene for generating the TCGA‐HCC subtypes based on the mRNA expression matrix by computing the importance of the gene in training a random forest (RF) model that classifies the HCC subtypes. The RF method was performed by the randomForest R package.^[^ ^55^ ^]^

### PPI Network and Pathway Based Functional Analysis of Subtype‐Relevant Genes

The interesting genes/proteins (the top‐ranked subtype‐relevant genes or the up‐regulated immune relevant proteins) were mapped into the human PPI network, and the connected sub‐network was extracted. Next, the optimal community structure of the sub‐network was identified based on the igraph R package, and nodes on the sub‐network were clustered into smaller network modules. Then, hypergeometric distribution based pathway enrichment analysis was applied on the members belonged to each network module to find the associated pathways.

### Prediction of the Two HCC Subtypes in the Other HCC Cohorts

The authors constructed a subtype predictor based on the TCGA‐HCC gene expression matrix of the top‐30 subtype relevant genes (see also Supporting Methods and Table S5, Supporting Information). This predictor, called GL1, was trained and tested by a lasso and elastic‐net regularized generalized linear model (glmnet R package).^[^ ^56^ ^]^

### ImmuP Calculation

Genes involved in immune system pathways of KEGG and showed significant up‐regulations in the C1 subtype than C2 (FDR <0.01, FC>1.5, Wilcox‐test, unpaired) were utilized as an immune relevant input gene list. Then, ImmuP was calculated based on the gene set variation analysis method^[^ ^57^ ^]^ which utilized the mRNA expression matrix of the TCGA‐HCC tumor samples to estimate the enrichment score of the input immune relevant gene list for each sample.

### Estimation of the Metabolite Accumulation Ability for the Poor Prognostic Subtype Based on the Directed MPI Network

For each metabolite, the authors extracted all its interaction proteins from the directed MPI network, and sorted out two types of proteins: 1) proteins with edges pointing to the metabolite, termed as UpPs; and 2) proteins with edges leaving the metabolite, termed as DownPs. Then, the relative accumulation score for this metabolite in the poor prognostic subtype can be estimated as: *deltaM* = *delta*(UpPs) − *delta*(DownPs), where *delta*(UpPs) or *delta*(DownPs) represents the sum of the log2FCs between the poor and well prognosis subtypes across UpPs/DownPs in mRNA level.

### MPI Prediction

**Step 1. Dataset preparation**. Both positive and negative datasets for MPIs were collected.

**Step 2. Feature description**. Six network association features were calculated to describe the correlations between one metabolite and one protein.

**Step3. MPI prediction model**. The authors employed RF to train and test the MPI prediction model.

See Supporting Methods, Supporting Information, for details.

### TME Analysis

The gene expression matrix for the TCGA‐HCC samples was utilized as the input data of web‐tool xCell.^[^ ^35^ ^]^ Meanwhile, the xCell tool also summarized the immune and stroma scores for each input sample. Tumor purity was predicted by the ESTIMATE^[^ ^58^ ^]^ method.

### Single Cell Data Analysis

Single cell RNA‐seq data for 5064 HCC T cells were downloaded from GEO (GSE98638).^[^ ^36^ ^]^ The authors applied the subtype predictor GL1 on the expression matrix of the HCC T cells, separated these cells into the C1/C2‐subtype like cells, and compared the expression levels of two representative proteins PDCD1 and CAT between the two subtype cells.

### Prediction of the Three HCC subtypes in the Other HCC cohorts

Based on the expression profiles of the C1 subtype HCC samples, the authors trained the second glmnet‐based subtype classifier GL2 to predict the sub‐population S1 and S2 in the C1 subtype samples. To annotate the three subtypes for the other HCC cohorts, they first applied the subtype predictor GL1 to separate the C1 and C2 subtypes, then they used the predictor GL2 on the C1 subtype samples to further identify the S1 and S2 subtypes.

### Statistical Analysis

Statistical and computational analyses and graph‐plotting were performed with R versions 3.6.3 and 3.5.0. Detailed information including the pre‐processing of data, data presentation, sample size, and statistical methods are reported in the corresponding experimental sections or figure legends.

## Conflict of Interest

The authors declare no conflict of interest.

## Supporting information

Supporting Information

Supporting Table 1

Supporting Table 2

## Data Availability

The data that support the findings of this study are available in GEO (https://www.ncbi.nlm.nih.gov/geo/, GSE14520, GSE54236, GSE15654, GSE76297, and GSE18494), TCGA pancancer atlas (https://gdc.cancer.gov/about‐data/publications/pancanatlas), ICGC (https://dcc.icgc.org/projects/LIRI‐JP), and the Supporting Information.
