# Supplementary material for: Identification and Characterization of Robust Hepatocellular Carcinoma Prognostic Subtypes Based on an Integrative Metabolite‐Protein Interaction Network
Source: Adv Sci (Weinh). 2021 Jul 11;8(17):2100311. doi: 10.1002/advs.202100311 (PMC8425875; doi:10.1002/advs.202100311)
Supplement: Supplementary file 1 — Supporting Information [file ADVS-8-2100311-s001.pdf]

© 2021 Wiley-VCH GmbH

ADVANCED SCIENCE
Open Access

Supporting Information

for *Adv. Sci.*, DOI: 10.1002/advs.202100311

Identification and Characterization of Robust Hepatocellular Carcinoma Prognostic Subtypes Based on an Integrative Metabolite-protein Interaction Network

*Di Chen, Yiran Zhang, Wen Wang, Huan Chen, Ting Ling, Renyu Yang, Yawei Wang, Chao Duan, Yu Liu, Xin Guo, Lei Fang, Wuguang Liu, Xiumei Liu, Jing Liu, Wuxiyar Otkur, Huan Qi, Xiaolong Liu, Tian Xia, Hong-Xu Liu and Hai-long Piao**

Supporting Information

Identification and characterization of robust hepatocellular carcinoma prognostic subtypes based on an integrative metabolite-protein interaction network

*Di Chen, Yiran Zhang, Wen Wang, Huan Chen, Ting Ling, Renyu Yang, Yawei Wang, Chao Duan, Yu Liu, Xin Guo, Lei Fang, Wuguang Liu, Xiumei Liu, Jing Liu, Wuxiyar Otkur, Huan Qi, Xiaolong Liu, Tian Xia, Hong-Xu Liu and Hai-long Piao**

Supporting Figures

Figure S1. Enlarged PCA plots. Four cancer types were extracted from Figure 2a separately, and the tumor and paired normal samples were represented by different colors. HCC: hepatocellular carcinoma; KIRC: Kidney Renal Clear Cell Carcinoma; BRCA: breast invasive carcinoma; LUAD: lung adenocarcinoma.

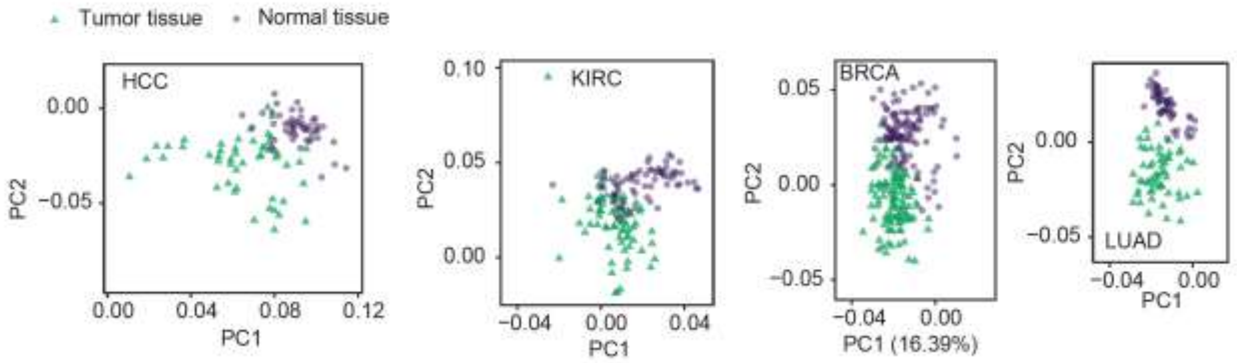

Figure S2. Clinical and molecular differences between the two MPI-based HCC subtypes. a-b) KM plot of the differential prognosis between the two subtypes in a GEO HCC dataset GSE14520 (a) and the other two cancer types KIRC and UCEC from TCGA (b). The two subtypes were determined by the MIPros-based PC1-PC2. For subtype C1, PC1-PC2 <0; for C2, PC1-PC2>0. c) Enriched pathways of each PPI network module among the top-ranked subtype-relevant genes. Each network module is named by "G_" plus one hub gene name. d) Enriched pathways of metabolites interacting with proteins from each PPI network module in (c). e) A sub-network of proteins with frequent interactions with the subtype-relevant genes in the PPI network. f) Volcano plot of the proteomic differences between the two TCGA-HCC subtypes. g) Enriched pathways of the up-regulated proteins (FDR<0.01, |FC|>0.2) identified in (f).

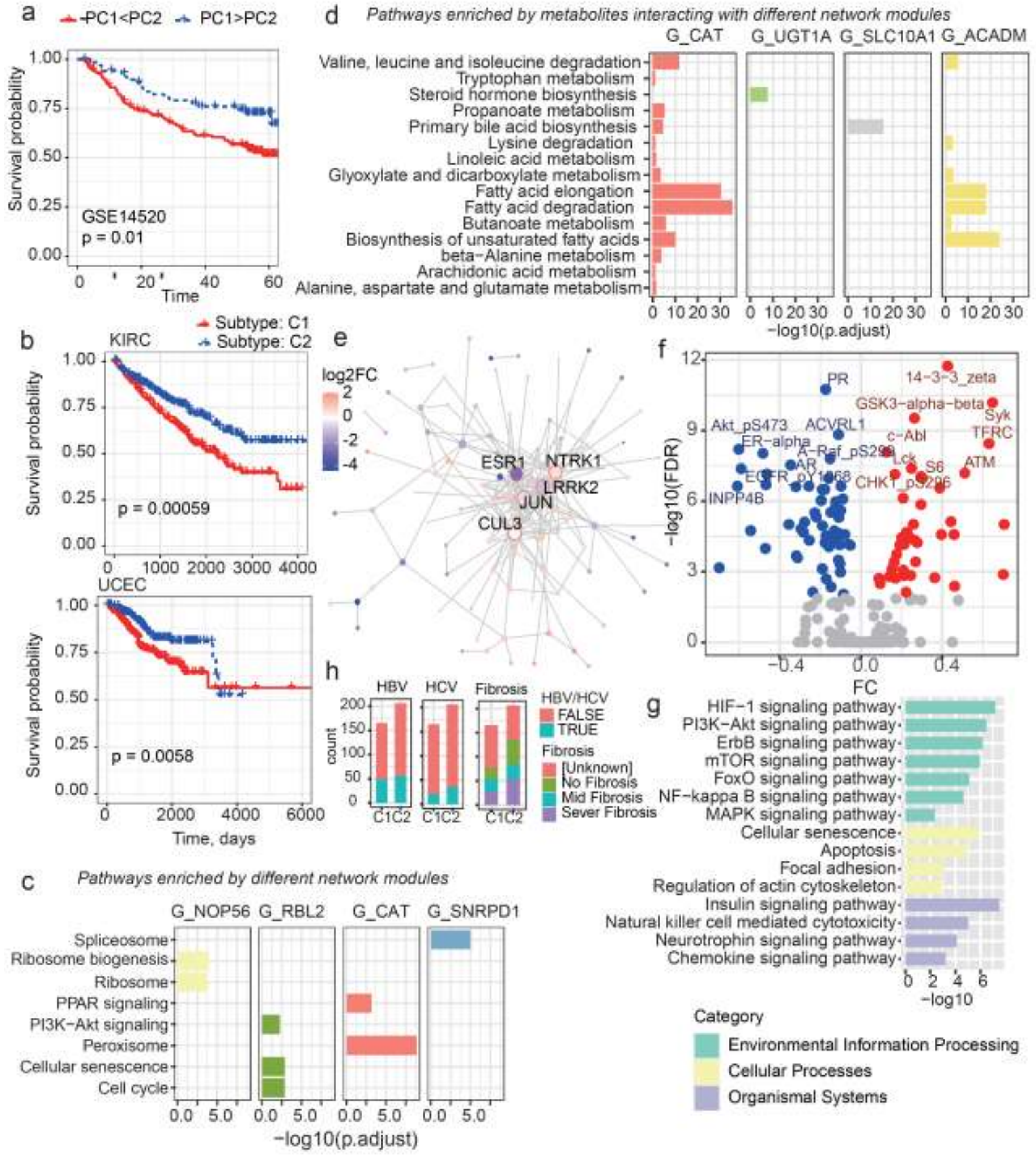

Figure S3. Molecular and clinical differences between the two HCC subtypes. a) DNA methylation based pathway level alterations between the two TCGA-HCC subtypes. Colors represent pathway categories. b-c) Boxplots of the DNA methylation levels of enzymes involved in lipid (b) and glucose (c) metabolism. (d) The sub-network of linoleic acid in the directed MPI network. Proteins are colored by the fold changes (FCs) in their mRNA expressions between the C1 and C2 subtypes in TCGA-HCC. e) Barplot showing the ability of the subtype C1 in ICGC-JP to accumulate (NES > 0) or consume (NES < 0) metabolites in different metabolism pathways in comparison with the subtype C2.

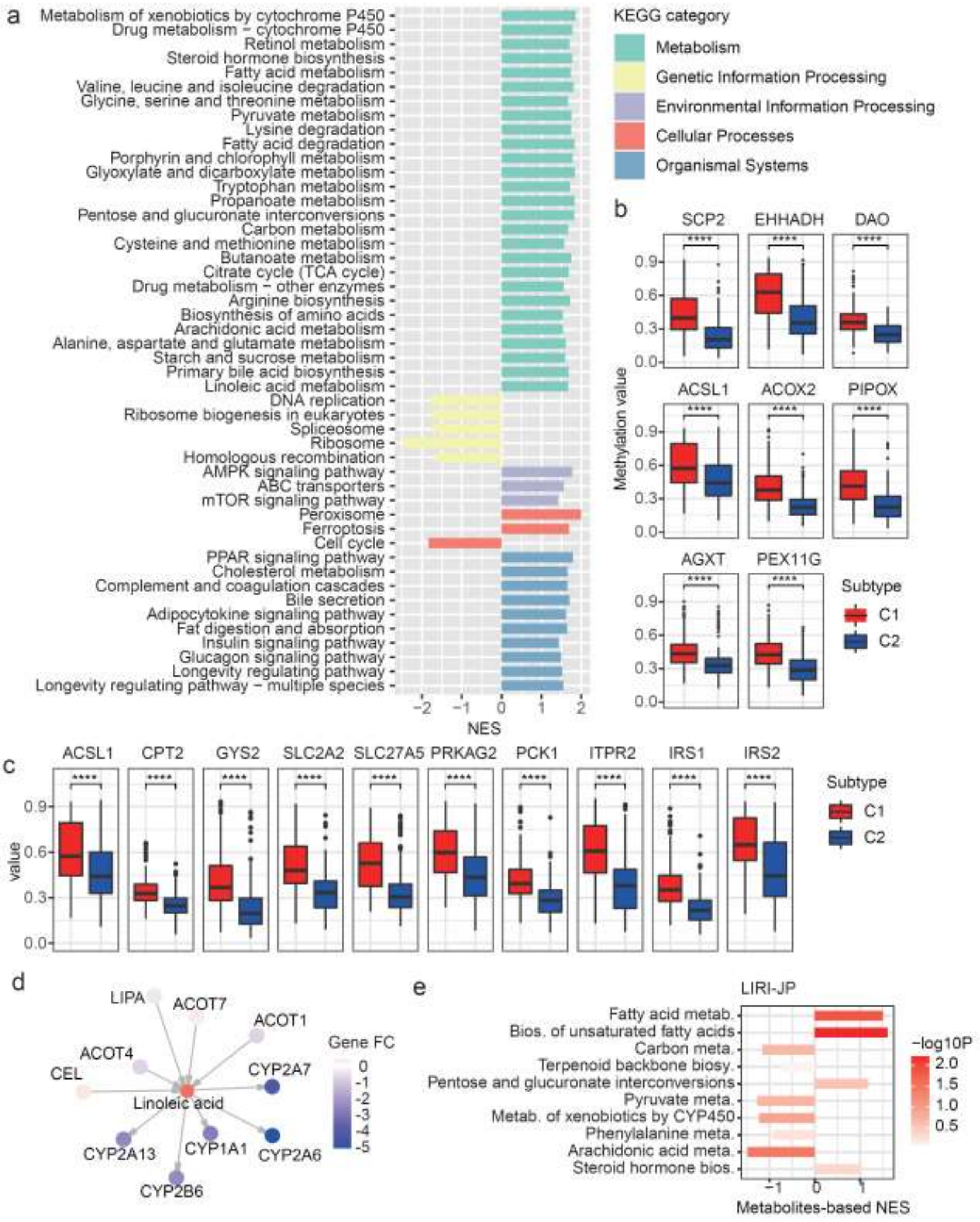

Figure S4. Metabolomics differences between the C1 and C2 subtypes identified in the GSE76279 HCC cohort. a) Volcanol plot of the metabolomics differences. The differences were tested by wilcox-test, unpaired, and the P values were adjusted by false discovery rate (FDR). Red and blue points represent the metabolites are significantly up or down regulated (FDR<0.05 and |FC|>2) in the C1 subtype. Grey points represent the differences are not significant. b) A MPI sub-graph of four metabolites involved in primary bile acid biosynthesis. After removing the reversible reactions, these four metabolites were only linked to an enzyme BAAT in the primary bile acid biosynthesis pathway, and their corresponding accumulation score deltaM were less than 0 (see also Methods, deltaM=deltaP-deltaS, since only one enzyme BAAT which can catalyze the production of these metabolites were left on the sub-graph, so deltaP = FC of BAAT, deltaS=0 here)

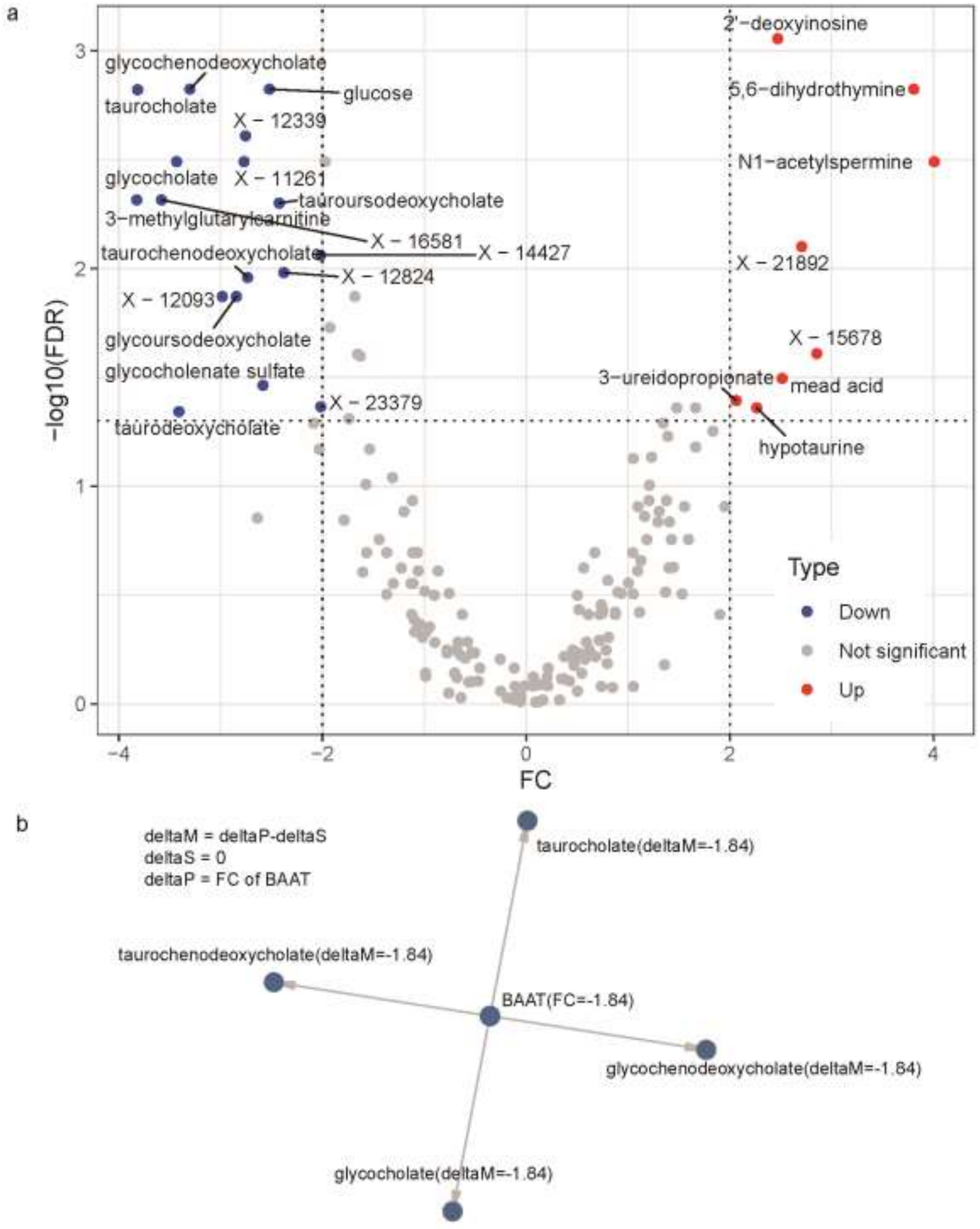

Figure S5. Interplay between metabolism and immune regulations. a) High confidential MPI prediction results for IKBKE. b) PPI sub-network about the immune-relevant proteins up-regulated in C1. Proteins are colored by the network modules. c) Pathways enriched by the members in the SRC-centered network module. d) The expressional differences in the immune-relevant proteins up-regulated in the subtype C1of four independent HCC cohorts.

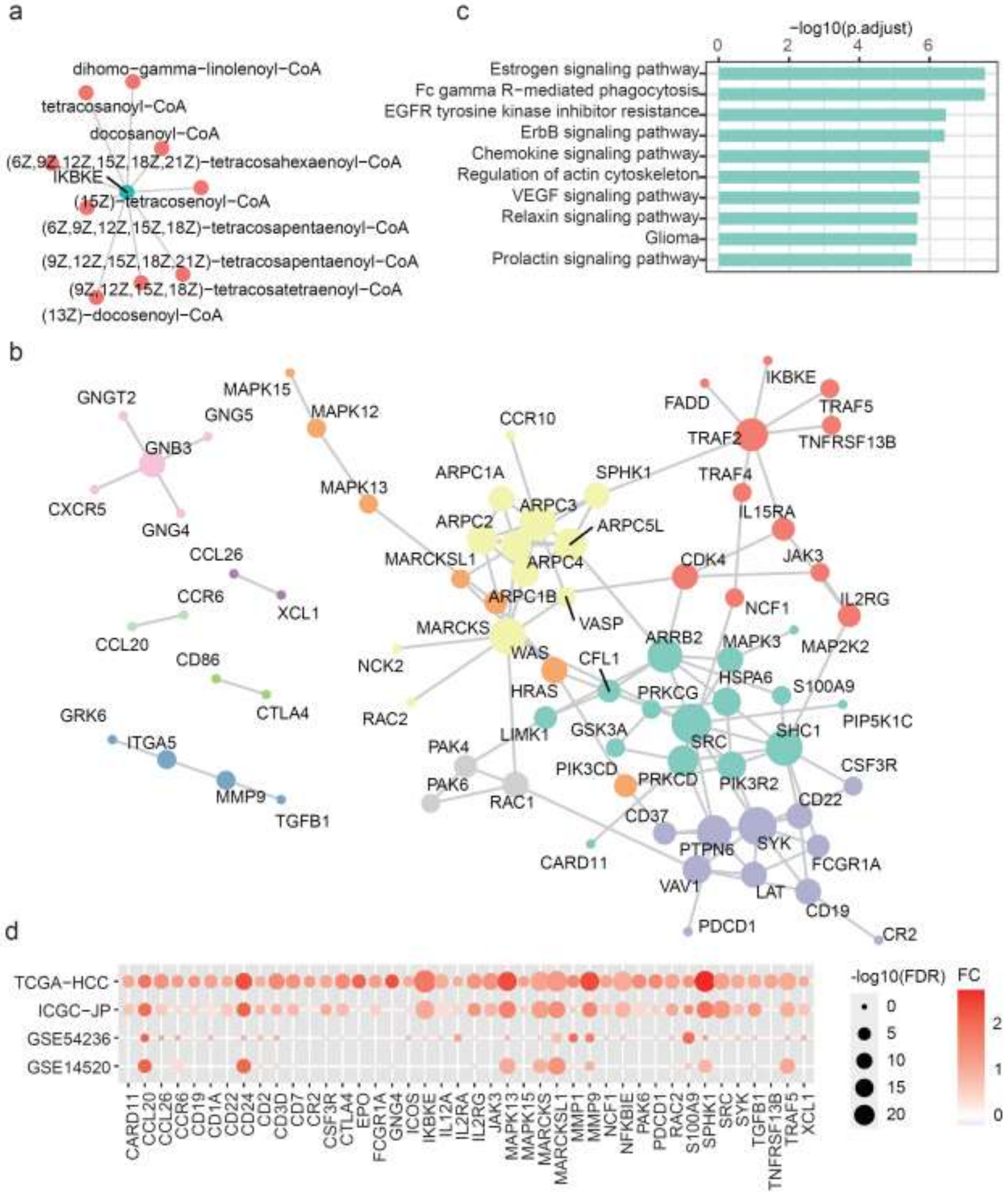

Figure S6. Further separation of the poor prognosis subtype in HCC into two subtypes based on TME compositions. a) Barplot of the GSEA-based pathway differences between the S1 and S2 subtype with respect to the mRNA expressions. b-c) Comparison with the Hoshida (b) and iCluster (c) subtypes in the TCGA-HCC dataset. The subtyping information was obtained by the TCGAbiolinks R package. d-e) KMplots of the survival rate differences among the Hoshida (d) or iCluster (e) based subtypes. f) KMplots of the survival rate differences among three further divided subtypes in the other three HCC cohorts. g) KMplots of the progression differences among three further divided subtypes in two HCC cohorts.

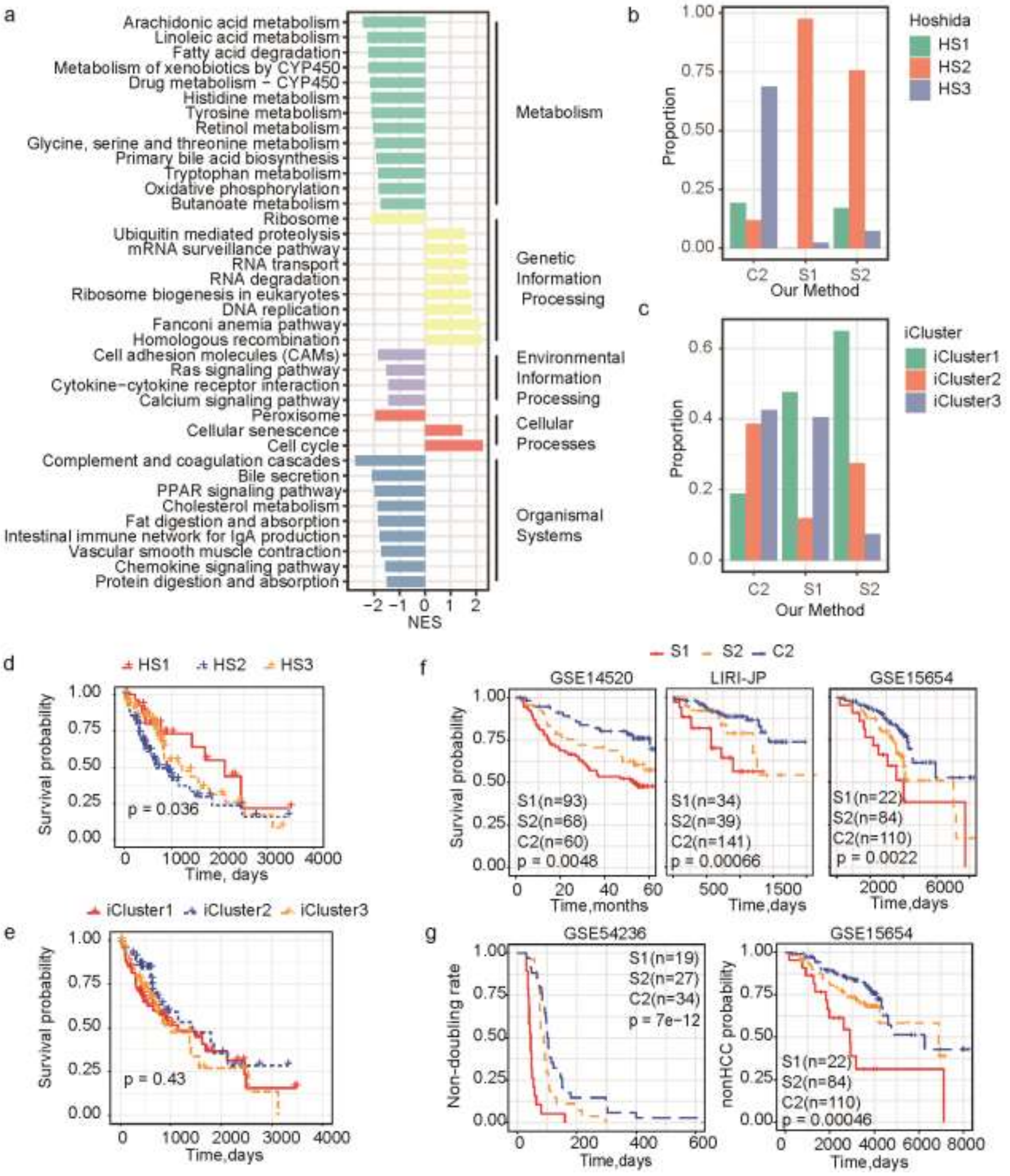

Figure S7. Validation of the two main HCC subtype features. a) Western blot analysis of HIF1α in SNU449 cells under normoxia or hypoxia conditions. Vinculin and GAPDH were used as loading controls. b) GSEA based immune system pathway differences between the SNU449 cells cultured under 6 hours of hypoxia and normoxia conditions. c) Heatmap of genes involved in Th1 and Th2 cell differentiation and showing higher expressions in the hypoxia SNU449 cells (T-test, one-sided). d) GSEA based metabolism or immune system pathway alterations of the HepG2 cell lines cultured after 12 hours of hypoxia treatment. e) Volcano plot showing the metabolite alterations of the SNU449 cell lines cultured after 6/24/48 hours of hypoxia treatment (T-test, two-sided, n= 6 for each condition).The red and blue points respectively represent up or down regulated genes in the hypoxia group.

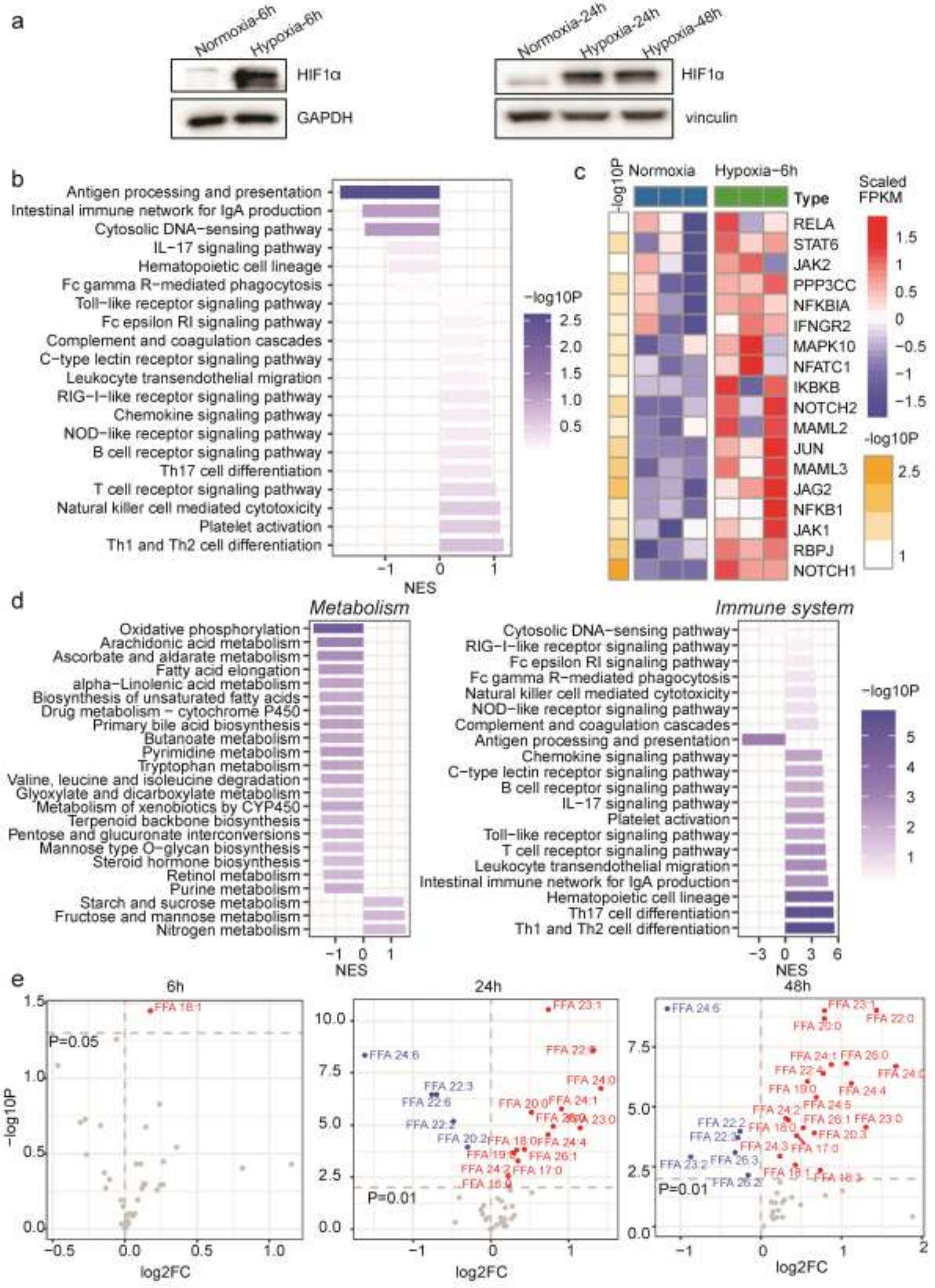

Supporting Tables

Table S1. Details about the MPI network.

Table S2. High-confidential MPI prediction results for proteins involved in immune system and up-regulated in C1.

Table S3. Detailed information of the free fatty acids (FFAs) in Figure 7.

FFA short name	Full name	Type
FFA 20:2	Eicosadienoic acid	Unsaturated
FFA 20:1	Eicosenoic acid	Unsaturated
FFA 20:4	Eicosatetraenoic acid (Arachidonic acid is the most common type)	Unsaturated
FFA 22:5	Docosapentaenoic acid	Unsaturated
FFA 24:3	Tetracosatrienoic acid	Unsaturated
FFA 24:5	Tetracosapentaenoic acid	Unsaturated
FFA 20:3	Eicosatrienoic acid	Unsaturated
FFA 22:4	Docosatetratenoic acid	Unsaturated
FFA 16:0	Palmitic acid/Hexadecanoic acid	Saturated
FFA 18:0	Stearic acid/Octadecanoic acid	Saturated
FFA 17:0	Heptadecanoic acid	Saturated
FFA 23:0	Tricosanoic acid	Saturated
FFA 19:0	Nonadecanoic acid	Saturated
FFA 20:0	Eicosanoic acid	Saturated
FFA 24:0	Tetracosanoic acid	Saturated
FFA 26:0	Hexacosanoic acid	Saturated
FFA 24:4	Tetracosatetraenoic acid	Unsaturated
FFA 24:2	Tetracosandienoic acid	Unsaturated
FFA 26:1	Hexacosenoic acid	Unsaturated
FFA 24:1	Tetracosenoic acid	Unsaturated
FFA 22:0	Docosanoic acid	Saturated
FFA 23:1	Tricosenoic Acid	Unsaturated
FFA 22:1	Docosenoic acid	Unsaturated
FFA 18:2	Octadecadienoic acid（Linoleic acid is the most common type）	Unsaturated
FFA 18:1	Octadecenoic acid	Unsaturated
FFA 18:3	Octadecatrienoic acid	Unsaturated

Table S4. The statistical results for significantly altered FFAs in the SNU449 cell lines cultured under 6/24/48 hours of hypoxia treatment comparing to normoxia treatment (T-test, two-sided, n= 6 for each condition). Only FFAs with p-value<0.05 for at least one time point were listed.

Name	6 hours		24 hours		48 hours	
	p-value	log2FC	p-value	log2FC	p-value	log2FC
FFA 10:0	0.320654	-0.26593	0.282321	0.12429	0.038741	0.197775
FFA 16:0	0.709037	-0.0124	0.008965	0.240134	0.014249	0.237824
FFA 17:0	0.537998	0.117914	0.000519	0.347579	0.000157	0.443758
FFA 18:0	0.055174	-0.05808	0.000159	0.331584	3.48E-05	0.339279
FFA 18:1	0.03561	0.176458	0.013916	0.249422	0.002618	0.428536
FFA 18:3	0.370151	0.109788	0.017633	0.5259	0.004382	0.737768
FFA 19:0	0.796924	0.026797	0.000217	0.293208	8.79E-07	0.577645
FFA 20:0	0.810705	0.012868	2.51E-06	0.522063	2.09E-09	0.787333
FFA 20:2	0.286437	0.357772	0.000115	-0.29934	0.094127	0.13116
FFA 20:3	0.357213	0.266102	0.18532	-0.10948	0.000122	0.662509
FFA 20:4	0.820596	0.055188	0.879664	0.016726	0.031543	1.012791
FFA 22:0	0.643663	-0.02844	2.56E-09	1.319741	9.34E-10	1.436896
FFA 22:2	0.99941	0.000211	6.55E-06	-0.48117	0.000103	-0.25952
FFA 22:3	0.551334	0.22202	3.50E-07	-0.69643	0.000193	-0.28661
FFA 22:4	0.924235	0.024891	0.092393	0.185478	4.07E-07	0.774133
FFA 22:5	0.913687	0.022965	0.345732	-0.06627	0.049244	0.801742
FFA 22:6	0.965579	0.010744	3.50E-07	-0.75947	0.377004	1.883725
FFA 23:0	0.88628	0.022217	1.38E-05	1.148743	6.95E-05	1.304242
FFA 23:1	0.148908	0.240965	2.84E-11	0.739014	9.71E-10	0.787864
FFA 23:2	NA	NA	0.030339	-0.40505	0.001217	-0.8728
FFA 24:0	0.469245	-0.10557	1.77E-07	1.414269	2.04E-07	1.678014
FFA 24:1	0.500483	0.131289	1.70E-06	0.907496	1.73E-07	0.875515
FFA 24:2	0.926159	-0.02241	0.002841	0.223401	3.01E-05	0.309977
FFA 24:3	NA	NA	0.033216	0.089068	0.001124	0.235159
FFA 24:4	NA	NA	2.88E-05	0.734682	1.06E-06	1.124056
FFA 24:5	0.501678	0.143251	0.045835	0.135121	4.05E-06	0.68764
FFA 24:6	0.195861	-0.30884	4.49E-09	-1.61912	8.07E-10	-1.16387
FFA 26:0	0.20527	-0.16947	1.14E-05	0.802146	1.55E-07	1.060213
FFA 26:1	0.423884	-0.14196	0.000146	0.431086	7.39E-05	0.525763
FFA 26:2	0.536245	-0.13214	0.07343	-0.09201	0.006985	-0.15915
FFA 26:3	NA	NA	0.116106	-0.12589	0.000791	-0.32113

NA: the FFA was not measured at the corresponding time point.

Table S5. The accuracies of the two HCC subtype predictors tested by 10-fold cross-over validation based on different numbers of top-ranked gene list.

Number of genes	Accuracy
Top-10	0.9433578
Top-20	0.9594476
Top-30	0.9731745
Top-40	0.9704757
Top-50	0.9707681

Supporting Methods

MPI network construction

Metabolism reactions in human were extracted from four resources including KEGG, Reactome, Human-GEM and BRENDA, where each reaction was composed of enzymes, substrates and products. Proteins (enzymes) and metabolites (both substrates and products) involved in the same reactions were combined pairwise as MPIs. KEGG reactions were obtained from KGML files (downloaded from https://www.kegg.jp/kegg/pathway.html, on 2019-03-08). Reactome reactions were parsed from SBML files (downloaded from http://reactome.ncpsb.org/download-data, on 2019-04-26). BRENDA reactions were from a summarized txt file (downloaded from https://www.brenda-enzymes.org/download_brenda_without_registration.php, on 2019-07-01). Human-GEM reactions were from xml and txt files (downloaded from https://github.com/SysBioChalmers/Human-GEM/tree/master/model, 2020-07-29). The direction between one substrate and one enzyme was from the substrate to the enzyme while that between one product and one enzyme was from the enzyme to the product, and the edges were bi-directional for the MPIs in reversible reactions. To unify the molecular identifiers from different resources, proteins and metabolites were respectively mapped into the unified gene symbols and KEGG compound IDs. Unduplicated MPIs from the four resources were integrated to construct a human MPI network.

PPI network construction

Human PPI data were downloaded from three PPI databases including BioGrid (https://downloads.thebiogrid.org/BioGRID, version BIOGRID-MV-Physical-3.5.183), MENTHA (http://mentha.uniroma2.it/download.php, version 20200413 for Homo sapiens) and IntAct (ftp://ftp.ebi.ac.uk/pub/databases/intact/current/psimitab, on 2020-04-13). Only PPIs belonged to human were retained. Proteins involved in these recorded PPIs from three databases were all mapped into the corresponding gene symbols, then unduplicated PPIs from these databases were integrated to construct the final human PPI network. This PPI network is un-directed.

Pathway information

Pathway information was obtained from KEGG. The KGML annotation files for all human pathways were parsed by the KEGGgraph R package, the genes as well as reactions in the pathways were extracted. Meanwhile, we also annotated the categories of the pathways according to the pathway catalogues in KEGG (https://www.kegg.jp/kegg/pathway.html#pk).

Genome-wide analysis of the pathway level differences between the two HCC subtypes

The mRNA expression differences between the two HCC subtypes were examined by Wilcox-test (un-paired) and log2FCs in terms of the mean mRNA expressions were calculated. Then, genes were ranked by the log2FCs, and the ranked gene list was utilized as the input for GSEA-based pathway enrichment analysis for all KEGG pathways.

Prognosis analysis for HCC subtypes

The prognosis differences among different HCC subtypes were examined by the Peto & Peto modification of the Gehan-Wilcoxon test, and the survival curves were fitted by the KM model.

Gene mutation analysis

The downloaded gene mutation data was in .maf format. MutSigCV-based significances of the mutations in the TCGA-HCC samples were downloaded from cBioportal. The mutated genes were ranked by q value (from smallest to biggest) and the top-50 genes were examined to see whether the mutations showed significant enrichment in one of the two TCGA-HCC subtypes based on hypergeometric distribution.

Proteomics analysis

We examined the protein level differences between the two subtypes based on the RPPA data (Wilcox-test, un-paired) and calculated the FC (difference value) between the two subtypes in terms of the mean protein level. The significantly up/down regulated proteins were identified, and their enriched pathways were examined by hypergeometric distribution.

DNA methylation analysis

We examined the DNA methylation level difference between the two TCGA-HCC subtypes for each gene (Wilcox-test, un-paired) and calculated the FC between two subtypes in terms of the mean methylation level. Then, genes were ranked by the FC, and the ranked gene list was utilized as the input for GSEA-based pathway enrichment analysis for all KEGG pathways.

Prediction of the two HCC subtypes in the other HCC cohorts

The genes were ranked based on their importance estimated by the random forest method in the part "Identification of subtype-relevant genes". Then, we try to construct subtype predictors based on the TCGA-HCC gene expression matrix of the top-ranked genes. Comparing the accuracies of the predictors based on different number of top-ranked genes (Table S3), we found that the top-30 based predictor performed the best. Consequently, we utilized the top30-based predictor to identify the subtypes.

MPI prediction

Step 1. Dataset preparation

Both positive and negative datasets for MPIs were needed to train a MPI prediction model. The positive dataset contained the MPIs (except those of metabolites with degree > 200) in the MPI network. The negative dataset contained the randomly combined metabolite and protein pairs, where the metabolites and proteins were the same as those involved in the positive dataset, and the random sampling was performed by the sample function in R. During the sampling process, we deleted the randomly combined pairs that happened to be a positive MPI, and retained the same number of negative metabolite-protein pairs as the positive dataset. Notably, during the prediction process, we ignored the direction of MPIs.

Step 2. Feature description

Six network association features were calculated to describe the correlations between a metabolite *a* and a protein *b*.

The first two features called NS1 and NS2 were based on the MPI and PPI networks (*G.MPI* and *G.PPI*). NS1 estimated whether the metabolite and the protein can directly interact with the same proteins, while NS2 estimated whether the metabolite and the protein can interact with the same proteins through one intermediary protein.

$$NS1(a,b) = \log 2(\left| Nei(G.MPI,a)\ \&\ Nei(G.PPI,b) \right| + 1)$$

where $Nei(G,i)$ means the neighbors of node *i* in the graph G, & means the interaction between two sets, |•| means the number of items in the set.

$$NS2(a,b) = \log 2(\sum_k TwoStepL(Nei_k(G.MPI,a),b,G.PPI) / \left| Nei(G.MPI,a) \right| + 1)$$

where $Nei_k(G,i)$ means the *k-th* neighbor of node *i*, and $twoStepL(k,b,G)$ means the number of two-step length paths between node *k* and node *b* in the network *G*.

NS3 examined whether the candidate protein P was similar with the known MIPros of the metabolite m considering the protein sequences.

$$NS3(a,b) = \sum_k SeqSim(Nei_k(G.MPI,a),b) / |Nei(G.MPI,a)|$$

where SeqSim means the protein sequence similarity (estimated by the twoSeqSim function in the protr R package [1])

NC1 and NC2 were based on a two-layer heterogeneous network (G.H, undirected) which contained metabolite-metabolite association (the first layer), PPIs (the second layer) and MPIs (links between layers). Two types of G.H were constructed, where in G.H1, metabolites involved in the same reactions were connected with each other, while in G.H2, structure similar metabolites were connected with each other. The metabolite structure similarity was calculated based on the Tanimoto coefficient between the atom pair descriptors of the metabolites (the ChemmineR package [2]).

$$NC1(a,b) = edgeC(a,b,G.H1)$$
$$NC2(a,b) = edgeC(a,b,G.H2)$$

Where *edgeC* calculated the edge connectivity, i.e., the minimum number of edges needed to remove to cut all paths from *a* to *b* on the network G.H1.

The last feature GS evaluated the GO-based functional similarity.

$$GS(a,b) = \max\{GOSim(Nei_k(G.MPI,a),b) \mid k \in (1, |Nei(G.MPI,a)|)\}$$

Where *GOSim*(*i*,*j*) calculated the sematic similarity (by GoSemSim R package [3]) between GO items of two proteins.

Notably, when calculating the features for a reported MPI, this MPI was removed temporarily from the MPI network, thus evading the over-representation of positive cases.

Step3. MPI prediction model

The six dimensional features were calculated for metabolite-protein pairs in both positive and negative datasets. Next, 10% of both the positive and negative datasets were retained for an independent validation, and the left 90% were utilized to train a MPI prediction model. We employed RF to train the MPI prediction model by the randomForest R package.[4] Finally, we used the trained RF model to predict positive probabilities for samples in the validation dataset, and evaluated the prediction performance by ROC.

CCLE data analysis

We downloaded the mRNA expression and drug response data of different cancer cell lines from CCLE (https://portals.broadinstitute.org/ccle/data), and we extracted the data for liver cancer cell lines. Then, we utilized GL1 to classify the cell lines into the corresponding subtypes, and compared the drug sensitivity between the two subtypes for each drug.

Experimental materials and chemicals

High performance liquid chromatography (HPLC)-grade methanol (MeOH), isopropanol (IPA), and acetonitrile (ACN) were purchased from Merck (Darmstadt, Germany). HPLC-grade chloroform was purchased from Duksan Pure Chemicals (Gyeonggi-do, South Korea). Ammonium acetate (AmAc), D-mannitol and free fatty acid (FFA) standards were from Sigma-Aldrich (St. Louis, USA). Ultrapure water was prepared by a Milli-Q system (Millipore, MA).

Transcriptomics profiling of cell lines

The SNU449 cell lines treated under normoxia and hypoxia for 6 hours were collected and their RNAs were isolated using TRIzol reagent. Sequencing libraries were constructed from total RNA using SMART-RNAseq Library Prep Kit (Hangzhou KaiTai, AT4201). In briefly, the mRNAs were isolated from total RNA with Sera-Mag Magnetic Olido (dT) particles, and then chemically fragmented. The fragmented RNA was reverse-transcribed into cDNA using random primer containing a tagging sequence at their 3'ends. Then, the cDNA libraries were amplified using the KAPA high-fidelity DNA polymer. Quality of the libraries was validated by the 2100 Bioanalyzer (Agilent Technologies). Subsequently, high-throughput sequencing was performed using a NovaSeq 6000 (Illumina) system. After sequences were mapped using hisat2 (version 4.8.2) against the Homo_sapiens.GRCh38.dna.primary_assembly.fa, the reads for each library were converted to FPKMs by Cuffdiff 2.1.137. RNA sequencing and library construction were

performed by technical staff at Hangzhou KaiTai Bio-lab.

Protein extraction and immunoblotting

For HIF-1α detection, culture medium was quickly removed and cells were immediately quenched in liquid nitrogen. Subsequently, cells were quickly scraped into tubes with 1× SDS-PAGE sample loading buffer. The primary antibodies including HIF-1α (1:1000, GeneTeX, GTX127309 or 1:1000, BD Biosciences, 610958), vinculin (1:1000, Santa Cruz, sc-73614) and GAPDH (1:1000, Proteintech, 60004-1-Ig) were used in this study.

Lipid extraction and fatty acids analysis

Cells used for fatty acids analysis were quickly washed with 5% D-mannitol-water (w/v) solution. Subsequently, cells were immediately quenched in liquid nitrogen. Extraction solvent was prepared by adding FFA standards to methanol, including d3-hexadecanoic acid (C16:0) and d3-octadecanoic acid (C18:0). Liquid-liquid extraction was performed as described previously[5] with slight modifications. In brief, cells were quickly scraped into EP tubes with 1 mL extraction solvent, followed by vortexing for 30 s. Then, 1 mL chloroform was added into the tubes and vortexed for 30 s, followed by adding 400 μL ultrapure water and vortexing for 30 s. After letting samples sit for 10 min at room temperature, the mixture was centrifuged for 15 min at 13000 × g, 4 °C. 600 μL lower phase was transferred for vacuum drying and stored at −80°C until analysis.

FFA profiling was carried out on ACQUITY™ Ultra Performance Liquid Chromatography (UPLC) system (Waters, Milford, MA, USA) coupled with AB Sciex tripleTOF 5600 plus mass spectrometer (Applied Biosystems Sciex, Foster City, CA, USA) as described before.[6] Briefly, samples were reconstituted in ACN/IPA/H2O (65:30:5, v/v/v/) containing 5 mM ammonium acetate, and 10 μL was injected into the LC-MS system and chromatographic separation was performed on a ACQUITY C8 column (2.1 mm × 100 mm × 1.7 μm, Waters, Milford, USA) maintained at 60 °C by gradient elution. Mobile phase

A was 60% ACN in water, and B was isopropanol:ACN (9:1), both containing 10 mM ammonium acetate. The flow rate was 0.3 mL/min, with the elution gradient as follows: 50% B was firstly maintained for 1.5 min, then linearly increased to 85% B from 1.5 to 9 min, then to 100% B at 9.1 and held for 1.9 min. The gradient was back to 50% B at 11.1 min and kept for 1.9 min to equilibrate column. For MS analysis, negative mode parameters were as follows: ion spray voltage, 4500 V; declustering potential, −100 V; collision energy, −10 V; and interface heater temperature of 550 °C. Curtain gas, GS1, and GS2 were set as 35, 55, and 55 psi, respectively. After data processing, fatty acids were assigned on the basis of MS/MS fragments, accurate masses, and retention time. The quantity of FFAs was normalized by total peak area.

Supporting references

[1] N. Xiao, D. S. Cao, M. F. Zhu, Q. S. Xu, *Bioinformatics* **2015**, *31*, 1857.
[2] Y. Cao, A. Charisi, L. C. Cheng, T. Jiang, T. Girke, *Bioinformatics* **2008**, *24*, 1733.
[3] G. C. Yu, F. Li, Y. D. Qin, X. C. Bo, Y. B. Wu, S. Q. Wang, *Bioinformatics* **2010**, *26,* 976.
[4] A. W. Liaw, M., *R News* **2002**, *2*, 18.
[5] M. Yan, H. Qi, T. Xia, X. Zhao, W. Wang, Z. Wang, C. Lu, Z. Ning, H. Chen, T. Li, D. S. Tekcham, X. Liu, J. Liu, D. Chen, X. Liu, G. Xu, H. L. Piao, *Metabolism* **2019**, 91, 18.
[6] Q. Xuan, F. Zheng, D. Yu, Y. Ouyang, X. Zhao, C. Hu, G. Xu, *Anal Bioanal Chem* **2020**, 412, 3585.
